# Network-level divergence in cyclic di-GMP signalling drives ecological versatility in *Acinetobacter baumannii*

**DOI:** 10.1038/s41522-026-00968-y

**Published:** 2026-04-09

**Authors:** Rubén de Dios, Valerie S. Forsyth, Lyuboslava G. Harkova, Kylie J. Schache, Brian Y. Hsueh, Hannah Strat, Brynn Riley, Alejandro Rubio, Antonio J. Pérez-Pulido, Christopher M. Waters, Harry L. T. Mobley, Sébastien Crépin, Ronan R. McCarthy

**Affiliations:** 1 Antimicrobial Innovations Centre, Department of Life Sciences, College of Health and Life Sciences, Brunel University of London, Uxbridge, UK; 2 National Biofilms Innovation Centre, School of Biological Sciences, Faculty of Environmental and Life Sciences, University of Southampton, Southampton, UK; 3 Department of Microbiology and Immunology, University of Michigan Medical School, Ann Arbor, Michigan USA; 4 Department of Microbiology, Genetics, and Immunology, Michigan State University, East Lansing, Michigan USA; 5 Centro Andaluz de Biología del Desarrollo (CABD-CSIC-JA), Universidad Pablo de Olavide, Sevilla, Spain; 6 Département de biologie, Faculté des Sciences, Université de Sherbrooke, Sherbrooke, Québec Canada; 7 Present Address: Human Health Therapeutics Research Centre, National Research Council Canada, Ottawa, Ontario Canada

**Keywords:** Computational biology and bioinformatics, Microbiology

## Abstract

Second messenger signalling pathways are known to play a fundamental role in governing bacterial physiology and mediating rapid adaptive responses to stimuli. Despite the extensive characterisation of second messenger signalling systems such as the cyclic di-GMP (c-di-GMP) signalling system in pathogens, relatively little is known about the role of these pathways in the priority pathogen *Acinetobacter baumannii*. To address this, we carried out a comprehensive exploration of c-di-GMP signalling across multiple modern *A. baumannii* clinical isolates. We elucidate this second messenger’s regulon and its role in biofilm formation and other virulence-associated behaviours. Furthermore, we demonstrate that specific enzymes controlling c-di-GMP levels are associated with specific international clones, with PdeD being identified as the primary functional phosphodiesterase in the worldwide prevalent International Clone I. Further characterisation of PdeD revealed it as a key regulatory node, controlling host colonisation in a murine model and persistence in the hospital environment. This indicates that the physiological control exerted by PdeD represented a distinctive advantage for the global dissemination of this lineage. Overall, this work uncovers the core regulatory role of c-di-GMP in underpinning the recalcitrant pathobiology of *A. baumannii* and also reveals PdeD as a potential novel therapeutic target.

## Introduction

Antimicrobial resistance (AMR) has been recognised by the World Health Organisation (WHO) as one of the most pressing concerns of our time and an important threat to human health^1,2^. Among the most threatening bacterial pathogens, multi-drug resistant (MDR) *Acinetobacter baumannii* stands as a major global health challenge. *A. baumannii* is a nosocomial pathogen that can produce a range of infections in immunocompromised patients, such as bacteraemia, pneumonia, wound infections and urinary tract infections^3–5^. It also causes recalcitrant outbreaks in healthcare settings due to its ability to form strong biofilms and resist disinfection treatments, combined with its outstanding tolerance to desiccation^6–8^. For these reasons, the WHO classified *A. baumannii* as a critical priority pathogen^9^.

*A. baumannii*’s “persist and resist” pathobiological strategy makes it difficult to eradicate once it colonises a host or an environment^5^. However, our understanding of the regulatory pathways that control *A. baumannii* pathogenesis lags behind other similarly challenging pathogens. Among such behaviours, biofilm formation (bacterial communities embedded in a polymeric matrix) is key to the capacity of this pathogen to tolerate environmental insults^10,11^. Importantly, biofilm formation correlates with a transition to chronic infection states within the host, becoming recalcitrant to the action of the immune system and antimicrobial treatments^12,13^.

Second messengers are a conserved mechanism through which bacteria can coordinate the simultaneous regulation of multiple phenotypes^14,15^. Second messengers are typically small nucleotide derivatives that act as ligands to activate or inhibit the function of their target proteins (i.e., effectors). Although there is a growing variety of bacterial second messengers, two well-studied examples of these signalling molecules are the antagonistic cyclic AMP (cAMP) and cyclic di-GMP (c-di-GMP)^14,15^. Whereas cAMP, synthesised by adenylate cyclases (ACs), typically promotes a motile lifestyle, c-di-GMP, produced by diguanylate cyclases (DGCs), is responsible for driving biofilm formation^12,16,17^. Both molecules can be degraded by specific phosphodiesterases (PDEs)^12,16,18^. In the clinical isolate *A. baumannii* AB5075, we have previously shown that cAMP, mainly synthesised by the AC CavA, plays an integral role in promoting motility and virulence to the detriment of biofilm formation^17^. On the other hand, the general effects of c-di-GMP in the lab reference strain *A. baumannii* ATCC 17978 show a positive regulation of biofilm formation^19,20^. Eight DGC enzymes (DgcA-F plus A1S_3345), along with two PDEs (PdeC and PdeE), have been identified in this strain^20,21^. Furthermore, three dual GGDEF/EAL domain-containing proteins with a predominant PDE activity (PdeA, PdeB and PdeD) complete the repertoire of enzymes involved in the synthesis/degradation of c-di-GMP in *A. baumannii* ATCC 17978^20,21^. Critically, this work on c-di-GMP regulation using this non-MDR low virulence type strain ATCC 17978^22^ is unlikely to reflect the behaviour of contemporary highly virulent MDR *A. baumannii* clinical isolates.

In this work, we elucidated the c-di-GMP regulon in two modern MDR clinical isolates of *A. baumannii*, and assessed the effect of c-di-GMP on their virulence-associated behaviours through manipulation of their c-di-GMP intracellular levels. We revealed that maintaining low levels of c-di-GMP is critical for the pathogenic success of this species in vivo. We also assessed the prevalence and distribution of DGC and PDE enzymes in a comprehensive *A. baumannii* phylogeny. Importantly, we identified PdeD as a major PDE enzyme restricting the c-di-GMP levels in *A. baumannii*. The deletion of *pdeD* affects key behaviours critical to the pathogenic success of *A. baumannii*, including capsule production, tolerance to disinfectants and desiccation, and resistance to host immune defences. We showed that PdeD prevents attachment to human epithelial cells whilst promoting intramacrophage survival. Finally, we demonstrated the in vivo relevance of these findings showing that a *pdeD* mutant has an impaired capacity to colonise the host in a murine model of bacteraemia. Overall, our results highlighted PdeD as a key element coordinating the pathobiology of *A. baumannii*.

## Results

### C-di-GMP induces global gene expression rearrangements in MDR *A. baumannii*

To understand the impact of c-di-GMP regulation on the physiology of contemporary MDR *A. baumannii* isolates, we used the genetically closely related strains AB5075 and AB0057 as models. Despite their similarity, they were sourced from different infection sites: a bone infection for AB5075 and a bacteraemic patient for AB0057^22,23^. To study the regulatory role of c-di-GMP without altering their native signalling networks, we designed a heterologous genetic system to manipulate its synthesis/degradation. We used the chromosomal neutral *att*Tn7 site to induce, via the IPTG-dependent *lacI*^*q*^*-Ptac* system, the expression of either a constitutively active version of the *Caulobacter crescentus* DGC pleD* to increase the c-di-GMP levels, or the *Pseudomonas aeruginosa* PDE *rocR* to decrease the c-di-GMP levels^24–26^. We previously demonstrated that this approach is effective for manipulating levels of c-di-GMP in AB5075^17^. Following the same design and validation, we observed a similar control of c-di-GMP levels in AB0057 (Supplementary Fig. S1). The relatively similar levels of c-di-GMP in the wild-type (WT) AB0057 compared to the derivative expressing *rocR* suggests that the c-di-GMP levels in our model clinical isolates are close to the minimum possible in the conditions tested.

**Fig. 1:**
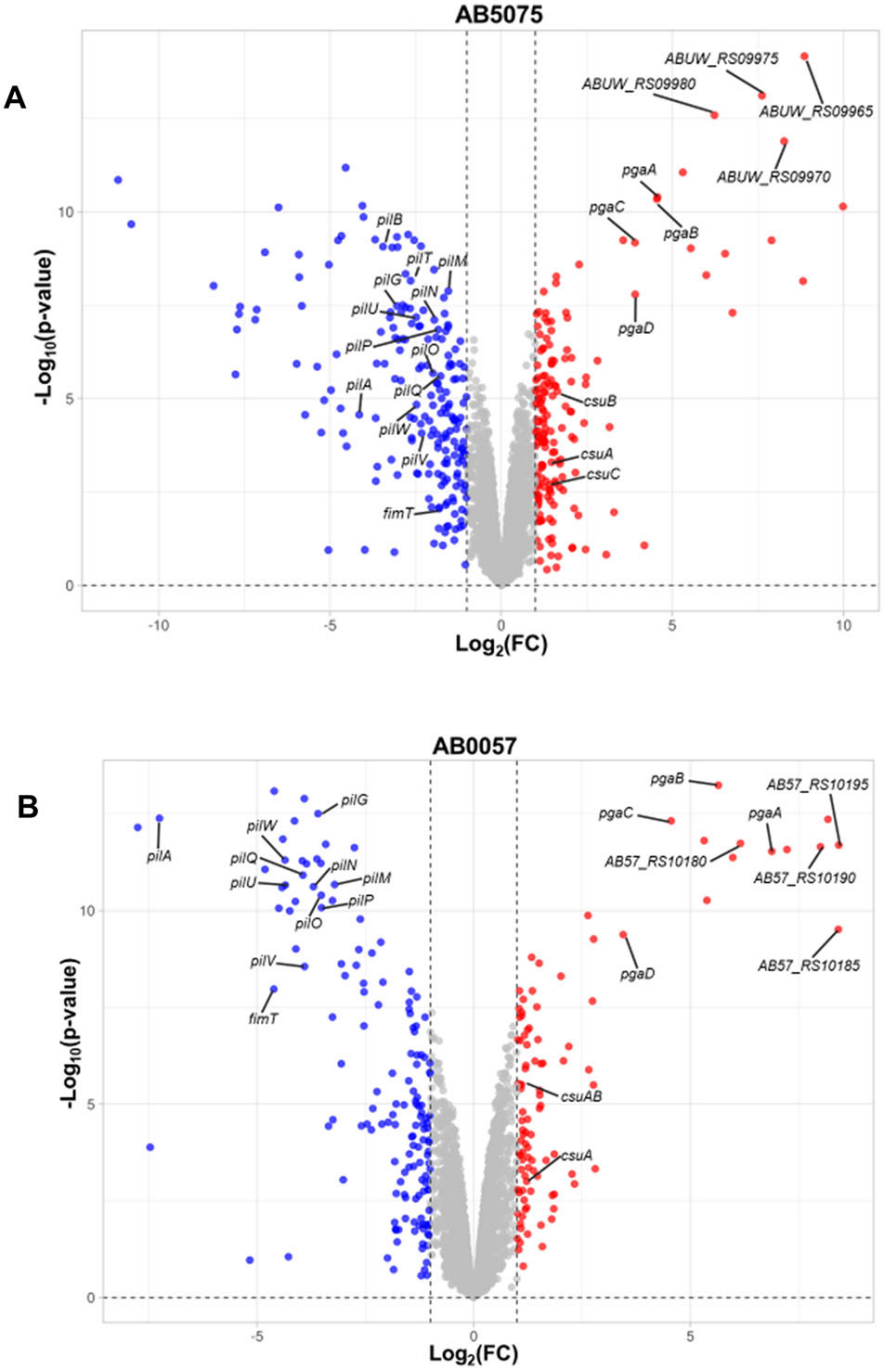
Heterologously increased c-di-GMP levels produce signature gene expression changes in the *A. baumannii* clinical isolates AB5075 and AB0057. Cultures of AB5075 (**A**) and AB0057 (**B**) with high c-di-GMP levels (expressing the DGC pleD*) versus low c-di-GMP levels (expressing the PDE *rocR*) were compared through dRNA-seq. Hallmark genes related to biofilm formation appeared significantly upregulated, whereas genes involved in twitching motility were significantly downregulated (Supplementary Table S1, Supplementary Table S2). Key genes for biofilm formation and motility significantly up- or downregulated appear labelled in the volcano plots. Positive Log_2_fold-change (Log_2_(FC)) values indicate an increased expression when c-di-GMP is elevated, whereas negative Log_2_(FC) values indicate a decreased expression when c-di-GMP is elevated. Three biological replicates of each strain were used for each compared sample group. Statistical analysis of differential gene expression was performed by edgeR’s exact test for comparing two groups of negative binomial counts.

To understand the breadth of transcriptional changes mediated by c-di-GMP in these MDR clinical isolates, we performed a differential RNA sequencing (dRNA-seq) experiment comparing cultures expressing pleD* with cultures expressing *rocR* (*i*.*e*., high vs. low c-di-GMP levels) for both AB5075 and AB0057. We observed that c-di-GMP significantly drives a differential regulation of 342 and 241 genes in AB5075 and AB0057, respectively (Fig. 1A, B, Supplementary Table S1, Supplementary Table S2). The analysis of the differentially regulated genes revealed that c-di-GMP affected the transcription of different gene functional groups related to metabolism, transport, cell adhesion, and, importantly, biofilm formation and motility, following the same trend for both strains. This included a general downregulation of the *pil* genes, encoding the type IV pili (T4P) machinery, which are involved in twitching motility and natural transformation^27,28^. On the other hand, the *pgaABCD* genes, involved in the production of the major structural matrix component poly-N-acetylglucosamine (PNAG)^29^, appeared highly upregulated in high c-di-GMP conditions. Interestingly, the Type I Csu pili coding genes, which have been previously reported as major biofilm formation determinants in *A. baumannii*^21^, appeared only slightly upregulated by c-di-GMP in both strains (1.4–1.6 log fold-change in AB5075 and 1.1–1.2 log fold-change in AB0057, with some of the *csu* genes below the differential expression threshold). This agreed with a mild downregulation of the phenylacetic acid degradation pathway, which has been shown previously to be oppositely regulated to the *csu* genes and to directly affect the regulation of these pili-encoding genes^30^. Additionally, another group of genes putatively encoding a fimbrial cluster (ABUW_RS09965-ABUW_RS09980 in AB5075 and AB57_RS10180-AB57_RS10195 in AB0057) were among the most upregulated genes in both datasets, suggesting that c-di-GMP preferentially targets these pili to promote biofilm formation in these strains. According to sequence similarity, this fimbrial cluster would be homologous to the previously described *cupABCD*/*abp2* chaperone-usher pili (CUP) cluster, which is critical for biofilm formation and host colonisation in the urinary tract clinical isolate *A. baumannii* UPAB1^31,32^. Overall, our results establish a link between c-di-GMP regulation and host colonisation through these pili.

We also specifically investigated potential enzyme-coding genes involved in the synthesis/degradation of c-di-GMP among the differentially regulated genes, whose expression may be responsive to changes in this second messenger. In both strains, we found that the DGC coding gene *dgcC* was significantly downregulated, suggesting a negative feedback loop in the c-di-GMP signalling pathway. Interestingly, the EAL-encoding gene *pdeE* was downregulated 0.56-log fold and 2.14-log fold in AB5075 and AB0057, respectively. This gene has been previously deemed catalytically inactive^19,20^, suggesting a c-di-GMP-dependent regulatory function to be elucidated.

Altogether, these findings show that c-di-GMP controls genes related to crucial behaviours for the interplay of *A. baumannii* with the environment and its virulence, including biofilm formation and motility. Furthermore, the c-di-GMP-dependent regulation of genes encoding GGDEF or EAL domains (*dgcC* and *pdeE*) highlights the presence of potential intrinsic regulatory feedback loops in the c-di-GMP signalling pathways of *A. baumannii*.

### C-di-GMP controls key behaviours defining the *A. baumannii* interplay between the environment and the host

**Fig. 2:**
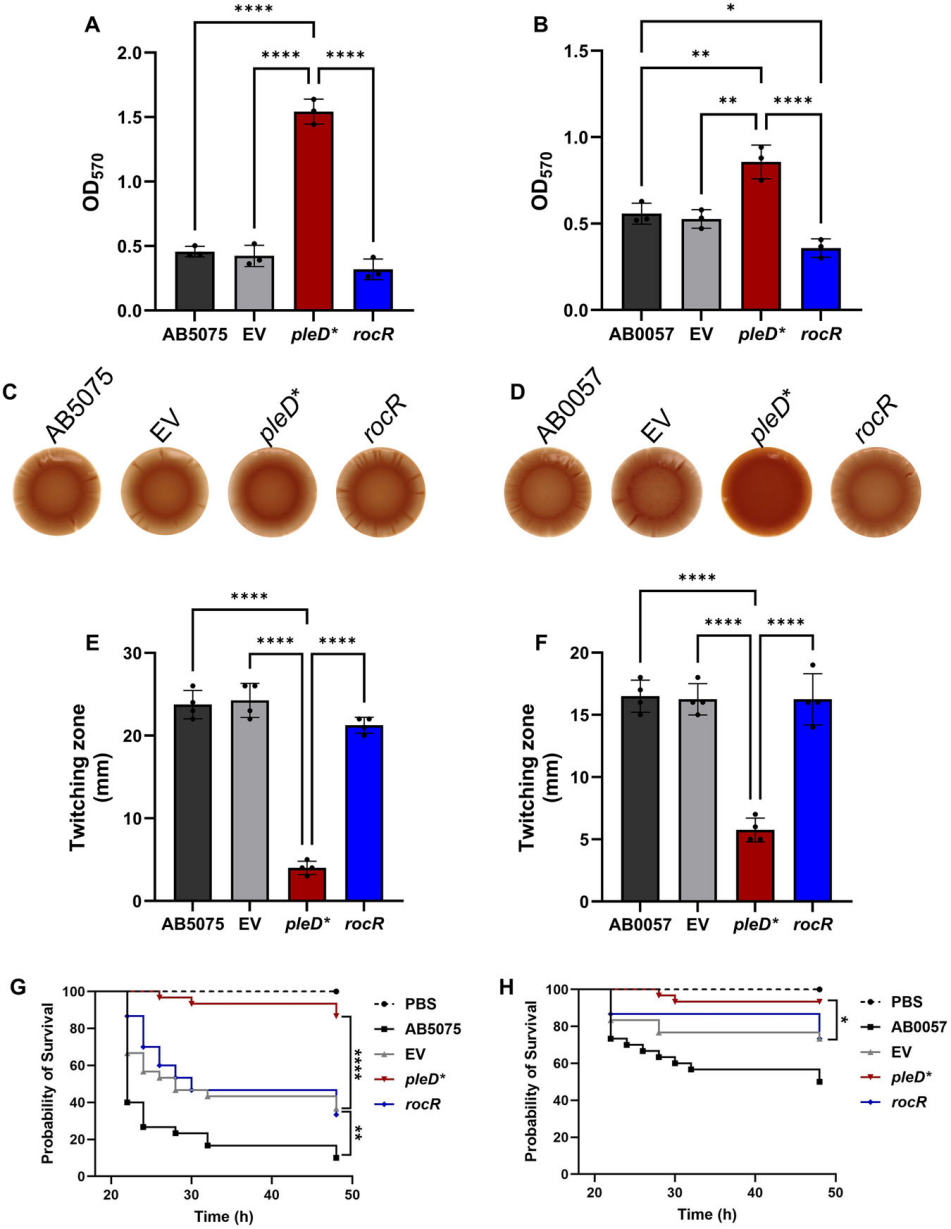
c-di-GMP controls key behaviours involved in the virulence of *A. baumannii* clinical isolates AB5075 and AB0057. Heterologous increase of c-di-GMP levels promoted biofilm formation in AB5075 (**A**) and AB0057 (**B**), whereas decrease of c-di-GMP levels trended to diminished biofilm formation. Averages ± S.D. from three biological replicates are represented. Statistical analysis was done by one-way ANOVA (Tukey’s correction). Similarly to the trend observed in the biofilm formation assay, a Congo red-based exopolysaccharide binding assay revealed a greater accumulation of the dye in colonies with high c-di-GMP levels in AB5075 (**C**) and AB0057 (**D**). The photographs shown are representative images of three biological replicates. Contrarily to biofilm formation, twitching motility was abolished under high c-di-GMP conditions for both AB5075 (**E**) and AB0057 (**F**), whereas a decrease in c-di-GMP levels did not promote motility further. Averages ±S.D. from four biological replicates are represented. Statistical analysis was performed by one-way ANOVA (Tukey’s correction). The effect of c-di-GMP on the virulence of AB5075 (**G**) and AB0057 (**H**) was assessed using the *G. mellonella* in vivo model. 30 larvae per sample group were injected with approximately 10^5^ cells of each WT strain or transconjugants bearing the pWH1266-Apr empty vector or vector derivatives constitutively expressing either pleD* or *rocR*. A control group was injected with an equivalent volume of PBS. Larvae survival was assessed from 22 to 32 h post-infection and at 48 h post-infection. The probability of survival was analysed with Log-rank (Mantel-Cox) test, indicating statistically significant differences for comparisons AB5075 vs. AB5075 (EV) (*p* < 0.01), AB5075 (EV) vs. AB5075 (pleD*) (*p* < 0.0001) and AB0057 (EV) vs. AB0057 (pleD*) (*p* < 0.05). Statistical significance is indicated as *p < 0.05; **p < 0.01; ****p < 0.0001.

Our transcriptomics analyses indicated that c-di-GMP controls the lifestyle switch between biofilm and planktonic growth in *A. baumannii* AB5075 and AB0057. Both the abilities to form biofilms and/or migrate across the milieu are important determinants of the bacterial interaction with the environment or a host^12^. However, in *A. baumannii*, the regulation of this transition has not been established. Hence, we next sought to experimentally validate the dependency of the biofilm lifestyle-associated phenotypes on c-di-GMP signalling. As a first step, we measured the biofilm formation capabilities of the different AB5075 and AB0057 derivatives expressing pleD* or *rocR* (high and low c-di-GMP levels, respectively) compared to an empty vector control (Fig. 2A, B). Compared to WT and empty vector controls, the strains with high c-di-GMP amounts reached significantly higher biofilm levels. On the other hand, expressing *rocR* trended to a slightly lower biofilm formation compared to the controls. This decrease was only statistically significant when comparing the WT AB0057 to its *rocR*-expressing derivative, which is consistent with our results presented in Supplementary Fig. S1 and by Harkova et al.^17^, showing a much smaller range to decrease c-di-GMP levels than increase. Directly related to biofilm formation is the production of an exopolysaccharide (EPS) matrix. The genes responsible for PNAG biosynthesis (*pgaABCD* operon) appeared upregulated by c-di-GMP in our dRNA-seq dataset. For this reason, we sought to determine whether the EPS production of AB5075 and AB0057 was directly impacted by c-di-GMP through a Congo red binding assay (Fig. 2C, D). We could distinguish subtle differences in dye accumulation comparing AB5075 expressing pleD* to its respective controls. However, an increase in the c-di-GMP levels in AB0057 led to a strong dye accumulation throughout the whole colony. On the other hand, in line with previous results, reduction of c-di-GMP levels did not significantly affect Congo red binding with respect to the control strains. Together, these results conclusively connect c-di-GMP signalling to the promotion of a biofilm lifestyle in *A. baumannii*.

Our dRNA-seq results also showed that the T4P machinery (*pil* genes) was impacted by c-di-GMP. As these bacterial appendages mediate twitching motility in *A. baumannii*^27,28,33,34^, we tested whether this phenotype was affected by c-di-GMP levels (Fig. 2E, F). C-di-GMP accumulation resulted in the abrogation of twitching motility compared to the control strains. In contrast, the expression of *rocR* did not produce significant differences in motility with respect to the controls, consistently with the results shown above for other phenotypes. This demonstrates that c-di-GMP not only promotes biofilm formation, but also directly impairs motility in *A. baumannii*.

Since both biofilm formation and motility contribute to *A. baumannii* pathobiology^5^, we aimed to test the direct effect of c-di-GMP on *A. baumannii* virulence using the *Galleria mellonella* acute model of infection. Since controlling the IPTG concentration to induce the expression of pleD* and rocR in vivo is challenging, we decided to constitutively express either pleD* or *rocR* from a non-repressed *P*_*tac*_ promoter from the replicative pWH1266-Apr plasmid in AB5075 and AB0057, and compare their effect to that of an empty plasmid control. AB5075 produced higher mortality rates compared to AB0057 (Fig. 2I, J), in agreement with previous literature^22^. RocR-mediated low c-di-GMP levels produced a similar larval mortality rate compared to the empty plasmid control, which itself led to reduced virulence compared to the respective WT strains. On the other hand, increasing the global c-di-GMP levels attenuated the virulence of both AB5075 and AB0057 when compared to their respective empty plasmid controls. These findings are consistent with a model where low c-di-GMP levels promote acute virulence. Overall, the results shown above point toward c-di-GMP as a central mediator of *A. baumannii*’s environmental persistence and pathogenesis^35^. Furthermore, they suggest that, under the conditions tested, *A. baumannii* keeps low basal levels of c-di-GMP because no DGC enzymes are active and/or through the action of its PDE enzymatic set.

### Network-level divergence in functional phosphodiesterase repertoire amongst International Clones

Our data indicate that, in the conditions tested, *A. baumannii* maintains a low concentration of c-di-GMP, thus promoting the planktonic lifestyle and sustaining virulence^12,13,35^. One hypothesis to explain this phenotype is that the endogenous PDE enzymes are constitutively active, thus keeping the global levels of this second messenger low. Hence, this would suggest that one or more of these PDEs may act as a central switch between environmental persistence and pathogenicity in *A. baumannii*. However, nothing is known about the conservation and functionality of PDEs and DGCs in contemporary MDR clinical isolates *A. baumannii*, such as AB5075 and AB0057.

**Fig. 3:**
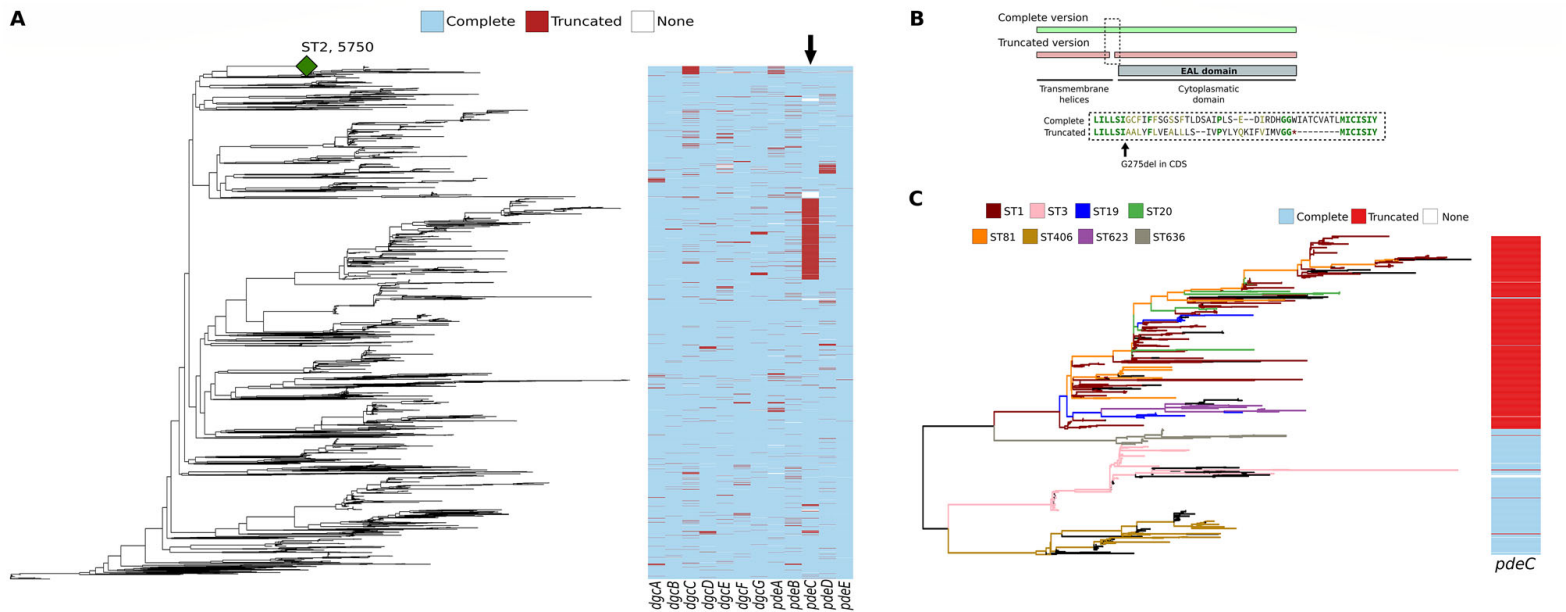
The evolutionary analysis of the *A. baumannii* DGC and PDE enzyme coding genes revealed a strong conservation, but the PDE PdeC appears truncated in a distinct phylogenetic group. **A** Molecular phylogeny of *A. baumannii* using 589 protein sequences that appeared encoded in all genomes. The metadata columns highlight genomes with complete (blue) and truncated (red) DGC and PDE coding genes. The case of the *pdeC* gene, which has a truncated sequence accumulation in one clade of the phylogeny, is marked with an arrow. The highly represented ST2 group, with 5750 genomes, has been collapsed in the interest of visual clarity, as it did not show any variations with respect to *pdeC*. **B** Schematic representation of the *pdeC* gene in strains AB5075 and AB0057. The functional and structural domains are highlighted. In addition, the sequence has been zoomed in, showing the detail of the frameshift occurring in its coding sequence. **C** Detail of the clade containing MLSTs with the truncated *pdeC* gene. The different MLST clonal groups are marked with different colours, as indicated in the panel. The metadata columns highlight genomes with complete (blue) and truncated (red) *pdeC* gene variants.

To comprehensively investigate the conservation of PDE and DGC coding genes in *A. baumannii*, we surveyed a curated pangenome of nearly 9000 *A. baumannii* genomes. We searched for all GGDEF and EAL domain coding genes (one or both), including *dgcA-F* (GGDEF-encoding), *pdeA*, *pdeB* and *pdeD* (dual GGDEF/EAL-encoding) and *pdeC* and *pdeE* (EAL-encoding), which were previously identified and named in *A. baumannii* ATCC 17978^19,36^. We also considered gene *A1S_3345*, an additional DGC coding gene identified by Guo et al.^21^, which we termed *dgcG* in agreement with the previously proposed nomenclature (locus tags of genes *dgcA-G* and *pdeA-E* are indicated in Supplementary Table S3). To gain additional evolutionary insights into the conservation of these genes, we assessed their conservation across *A. baumannii*’s molecular phylogeny according to their Multilocus Sequence Typing (MLST) clonal group classification. As a result, we determined that *dgcA-G* and *pdeA-E* can be considered as *A. baumannii* core genes, as they were identified in over 95% of the genomes analysed (Fig. 3A). Moreover, further variants of these genes, and additional genes containing GGDEF and/or EAL domains, appeared as accessory throughout the pangenome, with a percentage of prevalence between 1-95%. For example, some of these variants, such as the GGDEF-coding gene *BVG18_16310*, are encoded on mobile genetic elements, supporting the idea that plasmid-encoded DGCs facilitate the conjugal transfer of the carrier plasmid^37^.

In previous studies, the EAL-containing protein PdeC appeared as the main PDE, as it was the most catalytically active and its mutation or complementation produced greater phenotypic changes in vitro compared to other PDEs in *A. baumannii* ATCC 17978, which belongs to the phylogenetic group ST437 in the MLST scheme^19,20^. However, based on the current GenBank annotations of AB5075 and AB0057 (NZ_CP008706.1 and NC_011586.2, respectively), both belonging to the MLST1 group, *pdeC* appears as a pseudogene with a frameshift that introduces a premature stop codon upstream the EAL domain (Fig. 3B). To understand if the *pdeC* frameshift was specific to our two model strains or if it was more broadly conserved in the *A. baumannii* phylogeny, we screened all the *pdeC* variants present in the *A. baumannii* pangenome and clustered them according to the *A. baumanniii* MLST phylogeny. Interestingly, the truncated PdeC variants appeared encoded in closely related MLST groups (Fig. 3C), including MLST1 (one of the most widespread lineages of *A. baumannii* which includes AB5075 and AB0057^38,39^) with 99.2% prevalence, MLST19 (97.3% prevalence), MLST20 (100% prevalence), MLST81 (100% prevalence) and MLST623 (100% prevalence). Our results demonstrate that AB5075 and AB0057 can maintain low c-di-GMP levels in the absence of PdeC, indicating that another PDE enzyme must be responsible for restraining c-di-GMP levels.

### A heterologous expression screening reveals PdeD as an active PDE

**Fig. 4:**
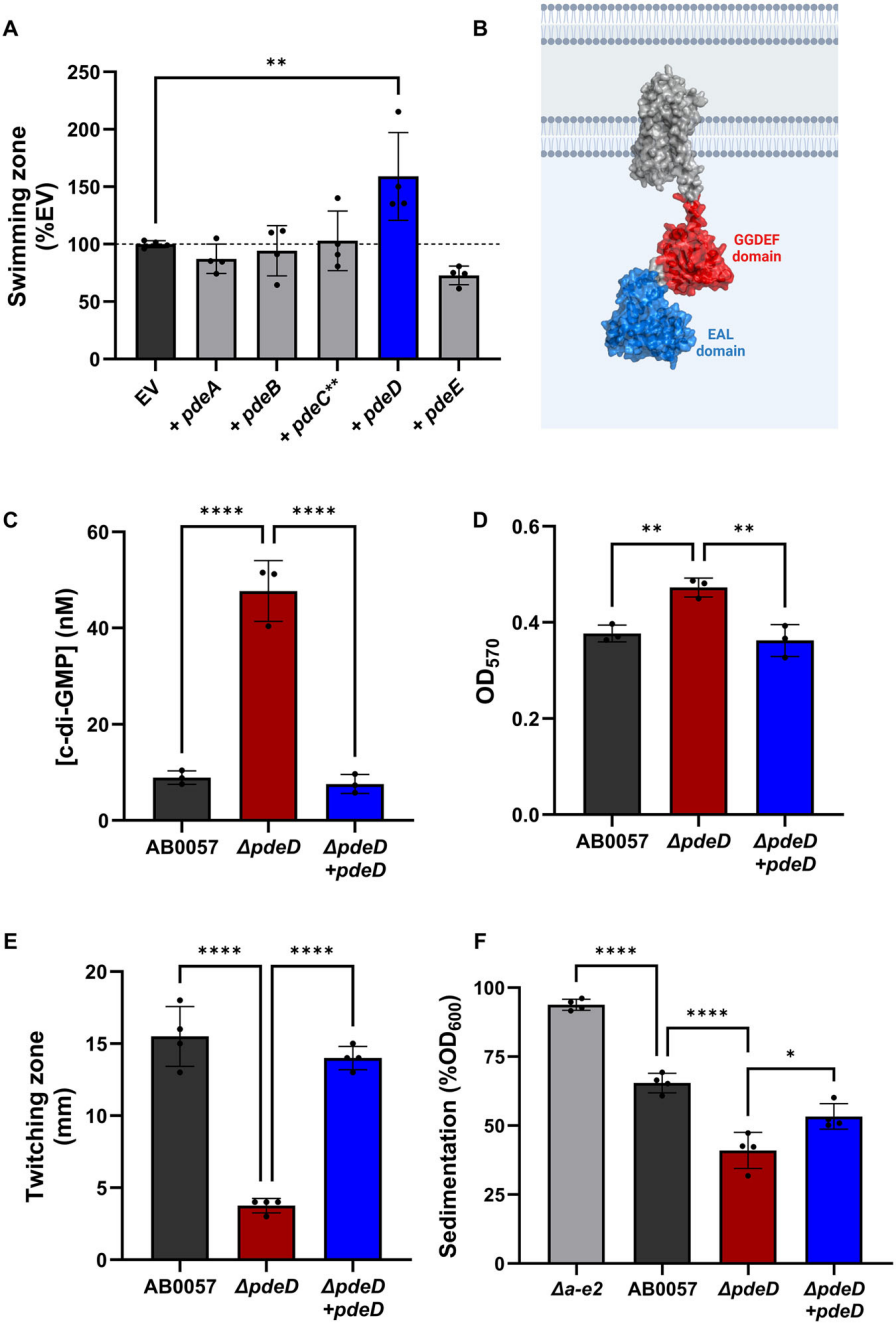
PdeD maintains low levels of c-di-GMP in *A. baumannii* AB0057, and impacts key virulence-associated behaviours. **A** Heterologous expression of individual *A. baumannii* PDE enzyme-coding genes revealed that only PdeD could decrease c-di-GMP levels enough to partially recover swimming motility in a swimming-defective, high c-di-GMP *E. coli* Δ*yhjH* mutant background. pdeC** indicates that this gene sequence encodes a truncated, non-functional PDE according to our results above (Fig. 3). Averages ± S.D. from four biological replicates are represented. Values are indicated as percentages with respect to the empty vector control (EV). Statistical analysis was done by one-way ANOVA (Dunnett’s correction). **B** The PdeD (ACJ40048.1) protein structure, based on the *A. baumannii* AB0057 reference genome (NC_011586.2) was modelled using AlphaFold^91^^,92^and visualised using PyMOL (Schrödinger and DeLano, 2020). The transmembrane domains are shown in grey, the GGDEF domain is indicated in red, and the EAL domain appears in blue. **C** The direct quantification of c-di-GMP levels in the Δ*pdeD* mutant compared to the WT AB0057 and the complemented mutant through UPLC/MS showed that this PDE enzyme maintains low levels of this second messenger. Averages ± S.D. from three biological replicates are represented. Statistical analysis was performed by one-way ANOVA (Tukey’s correction). **D** Due to the higher c-di-GMP levels, the Δ*pdeD* mutant showed increased biofilm formation levels compared to the WT AB0057 and complemented mutant. Averages ±S.D. from three biological replicates are represented. Statistical analysis was performed by one-way ANOVA (Tukey’s correction). **E** As a consequence of the higher c-di-GMP levels, the Δ*pdeD* mutant was impaired for twitching motility compared to the parental AB0057 strain and complemented mutant. Averages ± S.D. from four biological replicates are represented. Statistical analysis was performed by one-way ANOVA (Tukey’s correction). **F** A mucoviscosity assay revealed that the Δ*pdeD* mutant produced more capsule than the WT AB0057 and complemented mutant, which led to a decreased sedimentation. A non-capsulated Δ*gna-gne2* mutant (Δ*a-e2*) was used as negative control. Averages ± S.D. from four biological replicates are represented. Statistical analysis was performed by one-way ANOVA (Tukey’s correction). Statistical significance is indicated as *p < 0.05; **p < 0.01; ***p < 0.001; ****p < 0.0001.

Our heterologous manipulation of the c-di-GMP levels in *A. baumannii* AB5075 and AB0057 strongly indicated that these strains maintain low levels of this second messenger under the tested conditions. Similarly, the major PDE YhjH maintains low c-di-GMP levels and promotes swimming motility in *E. coli* under laboratory conditions^40,41^. Following an established screening procedure, we aimed to identify a potential constitutively active *A. baumannii* PDE that would revert the swimming-deficient phenotype of a Δ*yhjH E. coli* mutant^42^. To this end, we cloned and expressed all AB0057 EAL-domain coding genes (*pdeA*, *pdeB*, *pdeD*, and *pdeE*) from an arabinose-inducible promoter^43^, in a Δ*yhjH E. coli* mutant, and tested the swimming motility of the resulting strains (Fig. 4A). Since the PDE coding genes of AB0057 share 99–100% identity with their respective AB5075 orthologue, we only cloned those from AB0057. We also included *pdeC* in our analysis to validate its non-functionality. Our results showed that, among the different PDE coding genes expressed, only *pdeD* could partially restore the ability to swim of the Δ*yhjH* mutant compared to the empty vector control. PdeD is a GGDEF/EAL-containing protein with seven predicted transmembrane helices (Fig. 4B). Complementarily, a quantification of the c-di-GMP levels of the Δ*yhjH* mutant expressing *pdeD* by ultraperformance liquid chromatography-tandem mass spectrometry (UPLC-MS/MS) showed a trend to slightly lower amounts of the second messenger compared to the empty plasmid control (Supplementary Fig. S2A), which is similar to the results obtained when expressing *rocR* in *A. baumannii*. The remaining PDE coding genes tested, including *pdeC*, did not significantly affect the swimming capabilities of the *E*. *coli* Δ*yhjH* mutant. These results led us to the hypothesis that PdeD could play a central role in maintaining low levels of c-di-GMP, thus controlling the planktonic-to-biofilm lifestyle switch in *A. baumannii*.

### PdeD regulates virulence-associated behaviours in *A. baumannii*

To assess our hypothesis that PdeD is a major determinant in the pathobiology of *A. baumannii*, we created an in-frame Δ*pdeD* deletion mutant. The mutant was complemented by chromosomal integration of the *pdeD* gene under its native promoter at the *att*Tn7 site. Taking advantage of the genetic similarity between AB5075 and AB0057, and their parallel responses to c-di-GMP, we decided to focus our efforts on *A. baumannii* AB0057 as our model strain since it is more amenable to genetic manipulation^44^. Neither the Δ*pdeD* mutant nor its complemented derivative showed any growth defect in rich LB media or M9 minimal media with glycerol as carbon source, although we observed a slight, but not-significant, growth defect (-1.2-fold) in M9+glucose in the Δ*pdeD* mutant compared to the WT (Supplementary Fig. S3A–C). To confirm our hypothesis that PdeD is the main PDE in this strain, we determined c-di-GMP intracellular concentrations in the Δ*pdeD* mutant compared to the WT and the complemented strain by UPLC/MS and an ELISA-based c-di-GMP quantification. Coherently, we observed a significant increase in the levels of this second messenger in the mutant strain, with complementation restoring the WT levels (Fig. 4C, Supplementary Fig. S4).

We next assessed the contribution of *pdeD* in controlling c-di-GMP-associated phenotypes, such as biofilm formation and twitching motility. Previous studies, as well as our findings described above, indicated that biofilm formation and motility are antagonistically regulated^17,45,46^. As expected, the Δ*pdeD* mutant formed more biofilm and showed a greater Congo red accumulation than the parental strain, and complementation restored the WT phenotype (Fig. 4D and Supplementary Fig. S2B, respectively). On the other hand, twitching motility was completely abolished upon deletion of *pdeD*, with the complemented mutant recovering the WT levels of motility (Fig. 4E). Altogether, these results strongly indicate that PdeD alone can control the lifestyle balance in *A. baumannii* toward motile/planktonic growth rather than forming biofilm by limiting the levels of c-di-GMP in the cell.

As the capsule is a well-known virulence and fitness promoting trait in *A. baumannii*, and can contribute to biofilm development, we tested potential changes in capsular polysaccharide production in the Δ*pdeD* mutant compared to the WT and the complemented mutant using a silica colloidal density gradient (Supplementary Fig. S2C)^47–49^. Our results indicate a mild increase in capsular polysaccharide production in the absence of *pdeD*, and thus in a condition with higher c-di-GMP levels. To gain a better quantitative resolution, we then performed a mucoviscosity assay, conceptually similar to the colloidal gradients (a greater capsule production would decrease sedimentation), but measuring the turbidity of the cell suspension^50^. A Δ*gna-gne2* mutant was used as our non-capsulated control^46^. This further indicated that the Δ*pdeD* mutant produced significantly more capsular polysaccharides than the WT AB0057 and the complemented strain (Fig. 4F). These results were ultimately confirmed by profiling the capsular polysaccharide by SDS-PAGE, where a greater accumulation of bacterial capsule was visualised in the Δ*pdeD* mutant compared to the WT and the complemented strain (Supplementary Figure S2D). These results provide a conclusive link between c-di-GMP regulation and capsule production in *A. baumannii* for the first time. Interestingly, our dRNA-seq datasets did not show any differential transcription driven by c-di-GMP in genes involved in capsule production, which suggests that this regulation may occur at a post-transcriptional or a translational/post-translational level.

Altogether, our results demonstrate that the PdeD-mediated regulation of c-di-GMP levels controls behaviours associated with the recalcitrant biofilm lifestyle of *A. baumannii*.

### PdeD promotes tolerance to hospital environment-related stresses

**Fig. 5:**
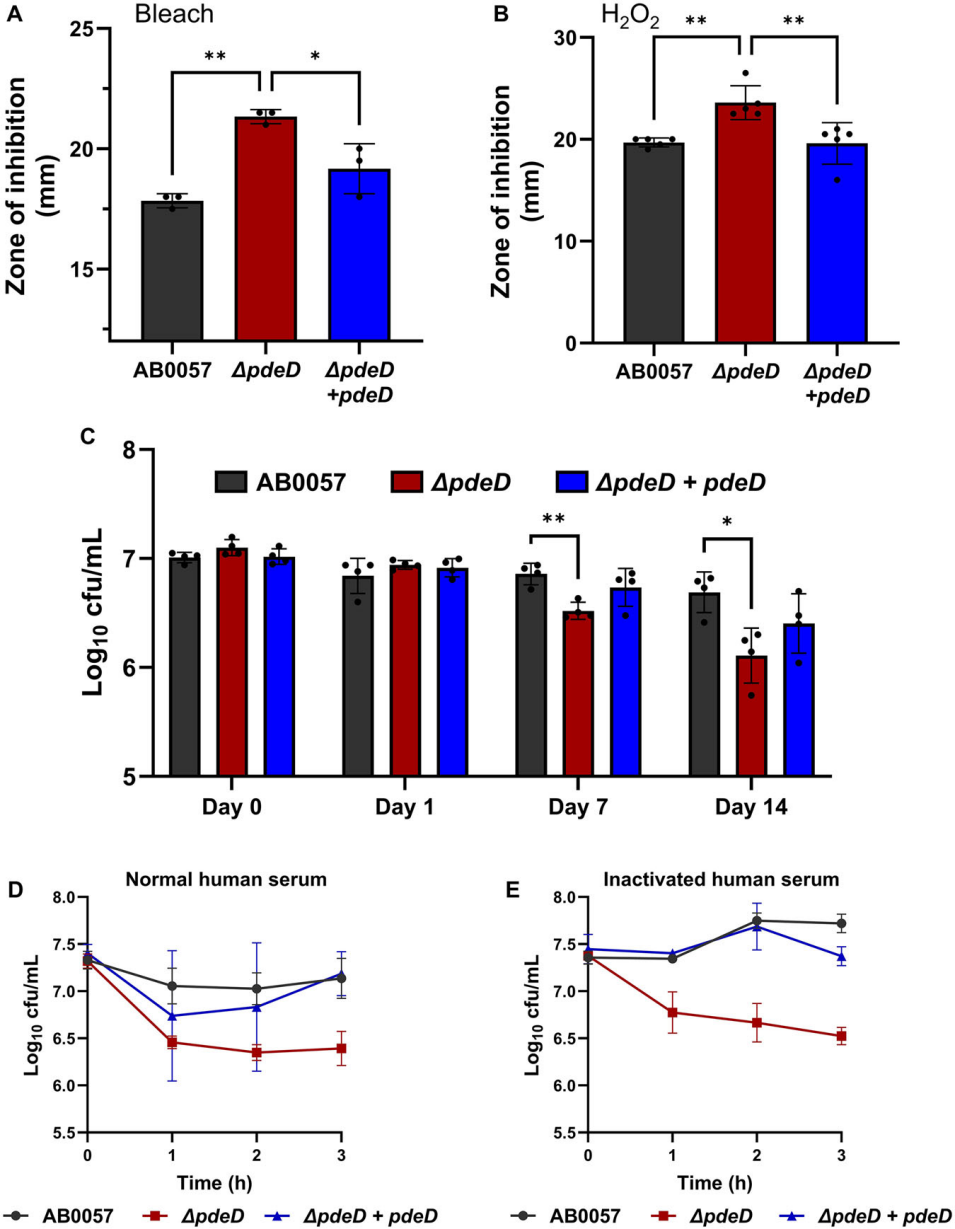
PdeD impacts the survival of *A. baumannii* AB0057 to hospital environment and host-related stresses. **A** The tolerance of the Δ*pdeD* mutant to various stresses related to the hospital environment was assessed. Compared to the WT AB0057 and the complemented mutant, the Δ*pdeD* mutant showed an increased sensitivity to 5% bleach (sodium hypochlorite) in a disc diffusion assay. Averages ±S.D. from three biological replicates are represented. Statistical analysis was done by one-way ANOVA (Tukey’s correction). **B** The Δ*pdeD* mutant was compared to the WT AB0057 and the complemented mutant regarding its sensitivity to 30% hydrogen peroxide (H_2_O_2_), which resulted in an increased sensitivity of the mutant against this oxidative agent. Averages ± S.D. from five biological replicates are presented. Statistical analysis was performed by one-way ANOVA (Tukey’s correction). **C** The survivability of the Δ*pdeD* mutant under desiccation was measured after 1, 7 and 14 days and compared to the WT AB0057 and the completed mutant, showing an impairment to withstand these conditions. Averages ± S.D. from four biological replicates are represented. Statistical analysis was done by repeated measures two-way ANOVA (Tukey’s correction). The survival of the Δ*pdeD* mutant to normal human serum (**D**) and fitness in heat-inactivated human serum (**E**) after 1, 2 and 3 h were also assessed and compared to the WT AB0057 and the complemented mutant, showing an increased sensitivity of the mutant in both conditions. Averages ± S.D. from four and biological replicates are represented in (**D** and **E**), respectively. Statistical analysis was performed by repeated measures two-way ANOVA (Tukey’s correction). For **D**, the comparisons between the Δ*pdeD* mutant vs. the WT AB0057 and the Δ*pdeD* mutant vs. the complemented mutant after 3 h resulted in statistically significant differences (*p* < 0.001 and *p* < 0.01, respectively). For **E**, the comparisons between the Δ*pdeD* mutant vs. the WT AB0057 and the Δ*pdeD* mutant vs. the complemented mutant after 3 h resulted in statistically significant differences (*p* < 0.05). For **A**–**C**, statistical significance is indicated as *p < 0.05; **p < 0.01.

*A. baumannii* is a nosocomial pathogen that causes extremely hard to eradicate outbreaks due to its ability to resist disinfection and to endure prolonged desiccation periods^7,8,51–54^. As these phenotypes are important features for *A. baumannii* to thrive in hospital settings, we sought to elucidate the possible role of PdeD, and thus c-di-GMP regulation, in controlling these tolerance behaviours. We first tested the role of PdeD in the resistance to a disinfectant frequently used in hospital settings such as hypochlorite (bleach), a strong oxidative agent. Performing a disc diffusion assay where the discs were saturated with 5% bleach, we measured the growth inhibition zone (diameter) following overnight incubation, where we observed that the mutant strain was more sensitive than the WT and the complemented strain (Fig. 5A). We also tested the role of PdeD in surviving disinfectants that disrupt the bacterial cell membrane, such as benzethonium chloride (BZT) and chlorhexidine gluconate (CHG). However, the Δ*pdeD* mutant showed an increased tolerance to these treatments compared to the WT and the complemented mutant strain (Supplementary Fig. S5A, B). This suggests that the effect of c-di-GMP regulation on disinfection tolerance may be determined by the mechanism of action of each treatment rather than providing a general protection.

As desiccation tolerance is associated with *A. baumannii* nosocomial outbreaks, we aimed to determine the potential connection between PdeD activity and this behaviour. We tested the ability of the Δ*pdeD* mutant, compared to the WT and the complemented mutant strain, to survive desiccation through 1, 7 and 14 days on a polystyrene plastic surface. Whereas there was no difference in survivability at day 1 post-desiccation between the Δ*pdeD* mutant and the WT, the mutant strain showed a significant reduction in the number of viable cells at day 7 and 14 (Fig. 5C). In both cases, reintroducing *pdeD* partially restored the desiccation tolerance of this mutant strain. Our results indicate that improper regulation of the c-di-GMP levels, *i*.*e*., accumulation via *pdeD* mutation, is detrimental to desiccation tolerance and demonstrate that controlling the production of c-di-GMP is capital for supporting this recalcitrant behaviour of *A. baumannii*, which is fundamental for its pathogenic success in the hospital-built environment.

### PdeD mediates resistance to innate host defences

It has been previously proposed that *A. baumannii* uses a virulence strategy based on resiliently enduring in the host, surviving defence responses and the rigors of the immune system^5^. As our previous results showed that an increase in c-di-GMP levels has a negative effect on the virulence of this pathogen in a *G. mellonella* model (Fig. 2G, H), we hypothesised that the PDE activity of PdeD could play a role in supporting this behaviour and protecting *A. baumannii* from the host-generated stresses. As one of the innate immune mechanisms, hydrogen peroxide production is a frontline defence against potential infective agents. Thus, we tested whether the Δ*pdeD* mutant was more susceptible than the WT and the complemented strain to this reactive oxygen species (H_2_O_2_) using a disc diffusion assay where the discs were saturated with 30% H_2_O_2_. As shown in Fig. 5B, the Δ*pdeD* mutant is impaired in the resistance to this oxidative agent, indicating that *pdeD* would play a role in maintaining the fitness of this pathogen in in vivo-related conditions. To further investigate the role of this PDE enzyme in resisting host-related stresses, we compared the ability of the Δ*pdeD* mutant to survive in 90% normal human serum (NHS) to that of the WT and complemented strains. Over the course of three hours, the mutant strain showed a 5.7-fold increased susceptibility compared to the WT and the complemented strains (Fig. 5D). This may mean that the Δ*pdeD* mutant has a decreased fitness in NHS or that it is less resistant to the complement proteins, the main NHS component responsible for the bactericidal activity. To elucidate this, the assay was repeated using heat-inactivated human serum, in which the complement was previously heat-inactivated. In these conditions, the Δ*pdeD* mutant continued to show a decreased survivability (15.5-fold) compared to the WT and the complemented mutant strain (Fig. 5E). Interestingly, these data suggest a fitness defect of the mutant strain in serum as opposed to an increased susceptibility to complement, as the number of CFU recovered at 3 h post-incubation for the mutant strain is similar in both conditions, i.e., 2.6 × 10^6^ (HNS) and 3.4 × 10^6^ (heat-inactivated). Altogether, these results indicate that the mutation of *pdeD*, and therefore the altered c-di-GMP accumulation renders *A. baumannii* unprotected against different host immune mechanisms in vitro.

### PdeD contributes to *A. baumannii* fitness in vivo

Our previous results using *G. mellonella* showed that c-di-GMP levels dictate the virulence behaviour of our two model *A. baumannii* clinical isolates in vivo (Fig. 2G, H). Furthermore, later results demonstrated that PdeD was involved in the resistance to different innate host defence mechanisms against potential pathogenic invaders (Fig. 5B, D, E). Accordingly, we sought to determine the contribution of *pdeD* in pathogenesis using cell culture-based assays and a murine model of bacteraemia, which is closely related to the native AB0057 site of infection.

**Fig. 6:**
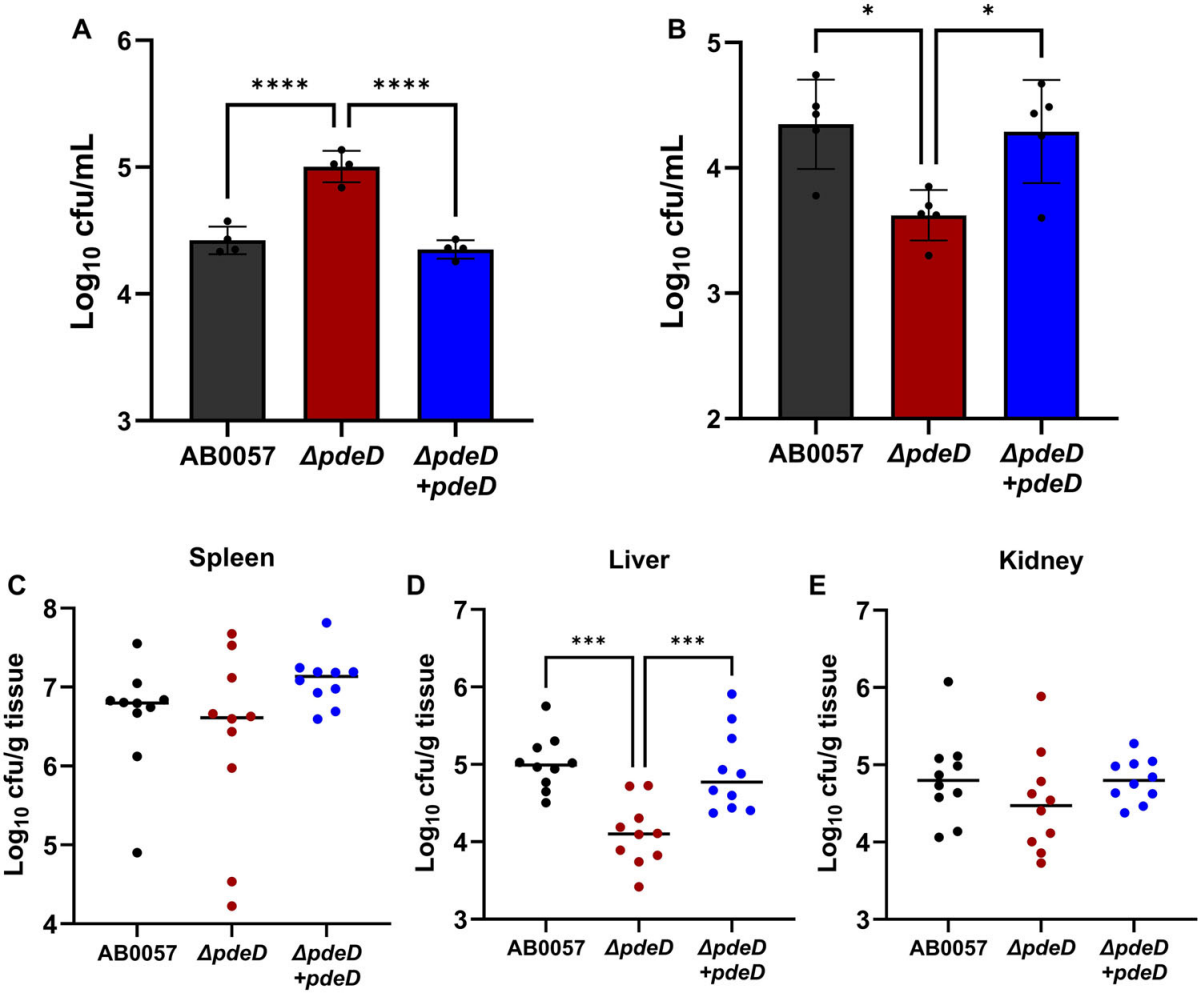
PdeD impacts the ability of *A. baumannii* AB0057 to interact with the host. **A** In agreement with its increased biofilm formation phenotype, the Δ*pdeD* mutant is more adherent to A549 human epithelial cells compared to the WT AB0057 and the complemented mutant after 2 h of co-incubation. Averages ± S.D. from four biological replicates are represented. Statistical analysis was performed by one-way ANOVA (Tukey’s correction). **B** The Δ*pdeD* mutant was impaired for intramacrophage survival at 30-min post-phagocytosis compared to the WT AB0057 and the complemented mutant. Averages ±S.D. from five biological replicates are represented. Statistical analysis was performed by one-way ANOVA (Tukey’s correction). The ability of Δ*pdeD* mutant with respect to the WT AB0057 and the complemented mutant to colonise spleen (**C**), liver (**D**) and kidneys (**E**) in a murine model of bacteraemia was also assessed 24 h post-infection. Dispersion and median from 10 individual mice in each group are presented. In **C**, the statistical analysis was performed using the Kruskal-Wallis test (Dunn’s correction) as at least one of the datasets did not pass the normality test. In **D** and **E**, the statistical analysis was done by one-way ANOVA (Tukey’s correction). Statistical significance is indicated as *p < 0.05; ***p < 0.001; ****p < 0.0001.

As we have shown above, PdeD negatively regulates the attachment of *A. baumannii* to plastic surfaces to form biofilms through control of c-di-GMP levels. Thus, we sought to elucidate whether PdeD would have the same effect on attachment to host cells. To test this, we co-incubated cell suspensions of the WT AB0057, the Δ*pdeD* mutant and the complemented mutant strain with A549 human lung epithelial cells and allowed them to interact for 2 h. Following the incubation, cell-attached bacteria were enumerated on LB-agar. Similar to the biofilm formation assay, we observed that the *pdeD* mutant is more adherent (3.8-fold) to A549 cells than the WT and the complemented strains (Fig. 6A). These results indicate that PdeD has a negative effect in the *A. baumannii* attachment both on biotic and abiotic surfaces via restricting the c-di-GMP level of this pathogen.

Another interaction that occurs between pathogens and the host immune system is the phagocytosis and digestion of bacterial invaders by macrophages. Modern *A. baumannii* isolates are capable of surviving and replicating in vacuoles after phagocytosis, which may be linked to their intrinsic ability to evade the immune system^53,54^. Therefore, the ability of this pathogen to survive in macrophage vacuoles during this process may determine the success of an infection. Thus, we tested whether PdeD could play a role in *A. baumannii* intramacrophage survival. The WT AB0057, the Δ*pdeD* mutant or the complemented mutant were incubated with RAW 264.7 murine macrophages, and at 30 min post-phagocytosis, the intramacrophage survival was determined via CFU enumeration on LB-agar. These results showed that the Δ*pdeD* mutant was 6.1-fold more susceptible to macrophage killing than the WT and the complemented strain (Fig. 6B).

*A. baumannii* AB0057 is an MDR strain isolated from the bloodstream^23^. To obtain an accurate view of the role of PdeD in the virulence of this strain, we tested the ability of the Δ*pdeD* mutant to colonise the host using our murine model of bacteraemia^44,55^. Mice were infected with each of the tested strains via tail-vein injection to induce bacteraemia, and the bacterial burden in the systemic organs (spleen, liver and kidneys) was quantified at 24 h post-infection. Although not statistically significant, we observed a trend in the Δ*pdeD* mutant to be less fit in the spleen and kidneys (Fig. 6C, E). However, in the liver, the mutant strain exhibited a 7.8-fold fitness defect compared to the WT strain, and complementation of the mutation restored the WT phenotype (Fig. 6D), suggesting a specific role of PdeD for establishing an infection in this organ. These results are also in alignment with our *G. mellonella* infections (Fig. 2G, H), as PdeD-mediated lower c-di-GMP levels tend to drive a more consistent colonisation of the host, whereas the absence of PdeD (hence higher c-di-GMP levels) seems to lead to a more unstable colonisation. Our in vivo results show that impairing the c-di-GMP regulation, via *pdeD* mutation, impacts the ability of *A. baumannii* to survive and colonise the host and highlight the importance of PdeD as a mediator of *A. baumannii* pathogenicity.

## Discussion

Over the last 40 years, c-di-GMP has moved from being a niche enzymatic co-factor to a universal bacterial second messenger signalling molecule governing a wide range of bacterial behaviours, such as motility, biofilm formation, and the cell cycle^56^. It has been shown to be an integral signalling system that enables bacteria the capacity to rapidly alter their global gene expression patterns and subsequent phenotypes in response to external microenvironment fluxes^12,57–59^. However, our understanding of c-di-GMP signalling in the WHO critical-priority pathogen *A. baumannii* is virtually non-existent, except for a few key revealing studies that recently started scratching the surface of c-di-GMP regulation in the lab-adapted reference strain *A. baumannii* ATCC 17978^19–21^. In this work, we aimed to provide the first comprehensive insight into the transcriptional and phenotypic responsiveness of *A. baumannii* clinical isolates to c-di-GMP changes and to identify the key c-di-GMP metabolising enzyme supporting the virulence strategy of this pathogen.

We first sought to explore the global regulon of c-di-GMP in two multidrug-resistant clinical isolates (AB5075 and AB0057) utilizing the well-characterised and functional non-native c-di-GMP modulating enzymes, the DGC pleD* and the PDE *rocR*, which increase and decrease the c-di-GMP levels, respectively^25,26^. Transcriptomic analyses revealed a common regulon with genes typically linked to multiple c-di-GMP-controlled phenotypes, such as adhesion (*csu* and the *cupABCD*/*abp2* homologous clusters), polysaccharide production (*pga*), and motility (*pil*) (Fig. 1A,B, Supplementary Table S1, Supplementary Table S2). Intriguingly, the gene clusters encoding the paralogous CUP cluster *prpABCD*/*abp1*^31,32^ (ABUW_RS11235-ABUW_RS11250 in AB5075 and AB57_RS08905-AB57_RS08920 in AB0057) did not appear differentially regulated by c-di-GMP (except for AB57_RS08920, with a -1.3-logfold differential expression in high c-di-GMP levels). This supports the functional differentiation between the two CUP clusters observed previously^32^ and suggests a role of c-di-GMP regulation in mediating this specialisation. In addition, our data show that c-di-GMP would preferentially regulate the *cupABCD*/*abp2* cluster over the *csu* pili, which so far have received most attention as mediators of *A. baumannii* biofilm formation^21,60–62^. We also uncovered that the transcription of the DGC *dgcC* and the PDE *pdeE* were downregulated at high c-di-GMP levels. This strongly suggests these are c-di-GMP responsive enzymes and form part of an intrinsic c-di-GMP control feedback loop to modulate the intracellular oscillations of this second messenger. This sort of regulatory circuitry helps maintain homoeostatic levels of this second messenger and fine-tunes the modulation of the transition between motile and sessile lifestyles^63^. To phenotypically validate the transcriptomic data, we confirmed that high c-di-GMP levels lead to increased biofilm formation and exopolysaccharide production, while abrogating twitching motility (Fig. 2). Furthermore, we determined that higher c-di-GMP levels attenuated the virulence of both strains in a *G. mellonella* acute model of infection (Fig. 2). This is consistent with the established paradigm that high c-di-GMP levels are required for infection chronification and pathogen persistence, whereas low levels of this second messenger are key for pathogen dissemination within the host and establishing acute infections^35^.

After exploring the c-di-GMP gene targets and phenotypic effects of this second messenger in MDR *A. baumannii* clinical isolates, we sought to delve into the native c-di-GMP signalling pathway of this pathogen. To this end, we used an evolutionary genomics approach to assess the prevalence and conservation of native PDE and DCG coding genes in *A. baumannii*. This revealed a core conservation of the genes involved in c-di-GMP production/degradation in this pathogen (Fig. 3A). Previous work had uncovered the EAL-containing protein PdeC as the most efficient active PDE in the reference strain *A. baumannii* ATCC 17978. However, our genomic analysis revealed that a distinct taxonomic group of the *A. baumannii* phylogeny, including MLST1 (International Clone 1), encodes a truncated version of PdeC that would abolish its activity. This conservation pattern suggests that, although PdeC is enzymatically more efficient under the conditions tested by Guo et al.^20^, it may be dispensable for the core c-di-GMP-mediated regulation of *A. baumannii*, particularly within the context of pathogenesis. Hence, we next aimed to identify which PDE, among the core repertoire of *A. baumannii*, could be responsible for restraining the c-di-GMP levels in our two contemporary clinical isolates. Through heterologous screening, we identified PdeD as a potential key regulator of the c-di-GMP levels in *A. baumannii*. Subsequent mutational and phenotypic analyses confirmed that PdeD controls intracellular c-di-GMP levels and influences a range of c-di-GMP-dependent phenotypes, including biofilm formation, EPS production, motility, and capsule production (Fig. 4, Supplementary Fig. S2).

A key factor in the emergence of *A. baumannii* as a challenging nosocomial pathogen is its ability to persist in hospital settings^8,51^. However, the potential contribution of c-di-GMP regulation to this recalcitrant behaviour, encompassing disinfectant resistance and desiccation tolerance, remained unexplored. Here, we demonstrate that the Δ*pdeD* mutant has an increased sensitivity to bleach (Fig. 5A), but a decreased susceptibility to the common disinfectants BZT and CHG (Supplementary Fig. S5). In the latter case, the results could be explained by the additional protection conferred by increased capsule production in this mutant^64^. Overall, these results connect the activity of PdeD to disinfection tolerance and highlight c-di-GMP, particularly through the action of this PDE, as a regulatory hub to facilitate specific, fast adaptive responses according to the type of environmental insults perceived. The ∆*pdeD* mutant also displayed an attenuated capacity for desiccation tolerance compared to the WT or complemented strains. This finding of decreased desiccation tolerance in a mutant, with an increased biofilm formation, contradicts the established model that increased biofilm is associated with increased desiccation tolerance^65^. This suggests that PdeD plays a novel role in the bidirectional regulation of these survival mechanisms.

As a final step in our study, we wanted to elucidate the impact of PdeD, and thus c-di-GMP levels, on the interplay of *A. baumannii* with the host and the immune defences. The ∆*pdeD* mutant displayed an increased susceptibility to reactive oxygen species and impaired fitness in human serum, including heat-inactivated serum (Fig. 5B, D, E). This suggests that, rather than playing a role in resisting the bactericidal activity of the complement, PdeD supports the fitness of *A. baumannii* when growing in human serum, which is relevant given the ability of *A. baumannii* to cause recalcitrant bloodstream infections. Subsequently, we demonstrated that PdeD plays a key role in regulating epithelial cell attachment and intramacrophage survival (Fig. 6A, B). These results indicate that PdeD-mediated low c-di-GMP levels determine the ability of *A. baumannii* to overcome a host cellular defence response such as macrophage-mediated phagocytosis. Recent studies have revealed that *A. baumannii*, and particularly modern clinical isolates, can survive and replicate inside macrophage vacuoles, which are proposed to act as a reservoir of this pathogen in the lungs^66–68^. In combination with our results, this suggests a potential role of PdeD and c-di-GMP in the modulation of this behaviour. Building on these findings, we used a mouse bacteraemia model to reveal that a *∆pdeD* mutant has a decreased capacity to colonise the host, although we could observe variations depending on the organ tested (Fig. 6C–E). This may suggest niche-specific adaptations of different *A. baumannii* strains that would lead to adaptations of their c-di-GMP signalling network.

The c-di-GMP signalling pathway has long been established as a key regulatory hub in bacteria, enabling cells to rapidly adjust their transcriptional landscape in response to external stimuli. Its importance in the context of infection has been dissected for a variety of pathogens, with *P. aeruginosa* being a paradigmatic model for c-di-GMP regulation^12^. However, our understanding of this signalling pathway in *A. baumannii* remains far from the level of resolution achieved for other similarly challenging pathogens. Despite most bacteria encoding multiple enzymes that can increase or decrease the intracellular levels of c-di-GMP, it has become increasingly apparent that specific enzymes within this repertoire have specific spatio-temporal roles, whereas others can act as central signalling hubs orchestrating the cellular programme. For example, mutations in the Wsp signalling systems in *P. aeruginosa* clinical isolates have been shown to constitutively activate the diguanylate cyclase WspR, driving cells toward higher levels of biofilm formation that define their pathogenic behaviours^69,70^. Another gap in our knowledge on the c-di-GMP regulation in *A. baumannii* compared to other pathogens is the known effectors of this second messenger, i.e., the proteins whose activity is modulated upon c-di-GMP binding. Currently, the protein A1S_2421, which contains a PilZ domain (widely recognised for binding c-di-GMP) and the elongation factor P are the only known c-di-GMP effectors in *A. baumannii*^20^. The vast number of genes that appear differentially regulated by c-di-GMP in our dRNA-seq datasets and the fact that capsule production is regulated by c-di-GMP at a level downstream of the transcription suggest the involvement of additional effectors that would execute the signals of this second messenger. These potential effectors remain to be elucidated and will be the focus of future research.

In summary, in this work, we have demonstrated that c-di-GMP controls a breadth of behaviours that are crucial for the pathogenic strategy of contemporary *A. baumannii* clinical isolates, including their persistence in the hospital-built environment and their interplay with the host. Importantly, we have identified PdeD as a key, widely conserved PDE enzyme that maintains *A. baumannii* in a “primed” low c-di-GMP state that is capital for host colonisation and the establishment of an acute infection. Altogether, this study provides a starting point to illuminate the full extent of the c-di-GMP signalling network in contemporary isolates of a current critical-priority pathogen, such as *A. baumannii*, and the role of this second messenger in its pathogenic success.

## Methods

### Bacterial strains and growth conditions

*A. baumannii* AB5075^22,71^, a kanamycin-sensitive AB0057 mutant^44^ and derivative strains, as well as *E. coli* strains were routinely grown in liquid or solid LB media (Miller) at 37 °C (180 rpm shaking when grown in broth). For *A. baumannii* strains, all cultures were started from pure stocks prepared with VIR-O colony morphotypes^72,73^. When needed for selection, media was supplemented with kanamycin (25–50 mg/L), amikacin (10 µg/ml), ampicillin (100 mg/L), apramycin (60 mg/L for *E. coli*, 30 and 100 mg/L for *A. baumannii*), gentamicin (50 mg/L), tetracycline (5 mg/L) or tellurite (6 mg/L for *E. coli*, 30 mg/L for *A. baumannii*).

A full list of the bacterial strains used in this work is shown in Supplementary Table S4. Details on plasmid and strain constructions are provided in the Supplementary Information Appendix.

### Plasmid construction

To alter the internal c-di-GMP levels in AB5075, miniTn7 transposon derivatives bearing the IPTG-inducible *lacI*^*q*^*-Ptac* expression system (pUC18T-miniTn7T-Tc-*lacI*^q^-Ptac^74^) and either the heterologous DGC coding gene pleD* (constitutively active)^25^ or the heterologous PDE coding gene *rocR*^26^ were used^17^. Given that AB0057 is naturally resistant to tetracycline, c-di-GMP levels were controlled in this strain following the same strategy but using pUC18T-miniTn7T-Tel-lacI^q^-Ptac^17^, which contains a tellurite resistance cassette instead of a tetracycline resistance gene, to clone pleD* and *rocR*. A DNA fragment containing the pleD* coding sequence plus a ribosome binding site was amplified from pMRB165^75^ with primers RBS fw PstI/pleD rv HindIII, digested with PstI and HindIII, and cloned into pUC18T-miniTn7T-Tel-*lacI*^q^-Ptac digested with the same enzymes, resulting in pUC18T-miniTn7T-Tel-*lac*I^q^-Ptac-pleD*. For the construction of pUC18T-miniTn7T-Tel-*lacI*^q^-Ptac-*rocR*, the *rocR* coding sequence was amplified from *P. aeruginosa* DSM 50071T^76^ using primers rocR RBS fw HindIII/rocR rv, digested with HindIII, and cloned into pUC18T-miniTn7T-Tel-*lacI*^q^-Ptac cut with HindIII and NruI.

As the expression of pleD* and *rocR* could not be controlled efficiently by IPTG induction in *G. mellonella* using the constructions described above, constitutively expressed alleles of pleD* and *rocR* under the *Ptac* promoter were cloned into the pWH1266-Apr plasmid^17^. The DNA fragments containing this promoter region and either pleD* or *rocR* were amplified using the primer pairs pre-Ptac/pleD rv HindIII or pre-Ptac/rocR rv, respectively, using the corresponding pUC18T-miniTn7T-Tc-*lacI*^q^-Ptac derivative construct as template. Subsequently, they were ligated into pWH1266-Apr, which had been cut with EcoRV.

To perform a functional screening following expression of EAL-domain containing genes in *E. coli*, the corresponding *pdeA, pdeB, pdeC, pdeD* and *pdeE* coding sequences (AB57_RS11625, AB57_RS14070, AB57_RS07335, AB57_RS03255, and AB57_RS14465, respectively, according to the AB0057 genome sequence GenBank record NC_011586.2) from AB0057 were cloned into the pBAD/myc-His A plasmid (pBAD; Invitrogen)^43^. Each gene was amplified by PCR using the primer pairs indicated in Supplementary Table S4. The resulting DNA products were digested with PstI and either PciI (*pdeD*) or NcoI (*pdeA, pdeB, pdeC* and *pdeE*) and were ligated into pBAD cut with the same enzymes as each insert to be cloned.

To generate an AB0057 derivative with a markerless in-frame deletion in *pdeD*, we followed a double recombination strategy using the plasmid pCVD442_MCS_Amk^44^. Approximately 1 kb size homologous regions upstream and downstream *pdeD* (including the initial and final codons) were joined together and cloned into pCVD442_MCS_Amk by Gibson assembly, generating pCVD442_MCS_Amk-Δ*pdeD*.

To complement the AB0057 ∆*pdeD* mutant, a DNA fragment containing the *pdeD* open reading frame and approximately 300 nucleotides upstream its start codon was cloned by Gibson assembly into pUC18T-mini-Tn7-Apra^77^, resulting in pTn7-Apra-*pdeD*.

All plasmid constructs were validated by restriction enzyme digest patterns and Sanger sequencing.

### Genetically modified *A. baumannii* strain construction

AB0057 derivative strains bearing inducible pleD* (miniTn7T-Tel-*lacI*^q^-Ptac-*pleD*) or *rocR* (miniTn7T-Tel-*lacI*^q^-Ptac-*rocR*) insertions to manipulate c-di-GMP levels were generated by four-parental mating as described by Harkova et al.^17^ with slight modifications, using pRK2013^78^ and pTNS2^79^ as helper plasmids. The conjugation spots on LB agar were incubated for 6 h at 30 °C, resuspended in PBS, and serial dilutions were plated on LB agar supplemented with 5 mg/L tetracycline and 6 mg/L tellurite and incubated overnight at 37 °C. Insertions were validated by PCR as previously described^74^.

To generate a ∆*pdeD* mutant in an AB0057 background, the pCVD442_MCS_Amk-*pdeD* plasmid was transferred to the WT AB0057 via biparental conjugation from the *E. coli* MGN-617 donor strain^80^ on LB agar plates supplemented with diaminopimelic acid (DAP, 50 mg/L). Transconjugants were selected on LB agar containing amikacin (without DAP). The selection of a second recombination event leading to the deletion of *pdeD* was achieved by culturing individual transconjugant colonies in LB broth for 2 h at 37 °C, diluting them, spreading on LB agar plates containing 10% sucrose, and incubating overnight at room temperature. Sucrose-resistant and amikacin-sensitive isolates were screened by PCR to confirm the *pdeD* in-frame deletion.

To complement the AB0057 ∆*pdeD* mutant, this strain was previously transformed with the pSTNSK plasmid, carrying the transposase *tnsABCD* that catalyses the miniTn7 transposition^81^. This resulting strain was conjugated with the pTn7-Apra-*pdeD* plasmid harboured in the MGN-617 donor strain. The conjugation mixture was incubated for 6 h at 30 °C on LB agar plates supplemented with 50 mg/L DAP. Following incubation, the mating spots were serially diluted, spread onto LB agar plates supplemented with 30 mg/L apramycin, and incubated at 37 °C overnight. Colonies were then screened by PCR for insertion of the miniTn7-based *pdeD* construct at the *att*Tn7 site as previously described^74^. Curing the thermo-sensitive plasmid pSTNSK was performed by successively passing the colonies on LB agar plates at 42 °C for 6 h, then overnight at 37 °C.

pWH1266-Apr-based constructions^17^, including the CensYBL-Ab biosensor, were introduced in the corresponding *A. baumannii* strains via triparental mating using pRK2013^78^ as helper plasmid. The conjugation mixtures were incubated on LB agar plates for 6 h at 30 °C and spread on LB agar supplemented with 100 mg/L ampicillin and 100 mg/L apramycin for selection.

### Fluorescent c-di-GMP quantification

Fluorescent quantification of c-di-GMP levels was measured using the biosensor CensYBL-Ab (and its inactive control CensYBL*-Ab) as described by Harkova et al.^17^. Briefly, overnight cultures of the tested strains bearing CensYBL-Ab (or CensYBL*-Ab inactive control) were diluted 1:100 in fresh LB broth and incubated at 37 °C, 180 rpm, for 1 h. After this, expression of the biosensor was induced by the addition of anhydrotetracycline (50 ng/ml), and cultures were further incubated for 2 h. Cells from 300 µl of each culture were pelleted by centrifugation and resuspended in 1 ml of PBS. 100 µl of each cell suspension (in technical triplicates) were loaded in a dark 96-well plate. YFP and mCherry fluorescence readings were taken using a CLARIOstar plate reader (BMG Labtech) using excitation/emission wavelengths of 570-15/620-20 nm and 497-15/540-20 nm, respectively. Cyclic-di-GMP levels were normalised by dividing the YFP fluorescence readings (c-di-GMP level dependent) by the mCherry fluorescence readings (constant and proportional to cell density).

### Differential RNA sequencing

Three independent overnight cultures of AB5075 carrying either the miniTn7-Tc-*lacI*^q^-Ptac-pleD* or the miniTn7-Tc-*lacI*^q^-Ptac-*rocR* insertions and AB0057 carrying either the miniTn7-Tel-*lacI*^q^-Ptac-pleD* or the miniTn7-Tel-*lacI*^q^-Ptac-*rocR* insertions were diluted to OD_600_ 0.05 in LB broth supplemented with 1 mM IPTG. The cultures were incubated at 37 °C and shaken at 180 rpm until they reached mid-log phase (OD_600_ 0.6–0.7). Then, cells from 1 ml of each culture were pelleted and washed with RNAlater. The RNA was isolated using the RNAeasy Kit with on-column DNAse digestion (Qiagen). The RNA integrity was assessed using a Bioanalyzer (Agilent 2100 Bioanalyzer and Agilent RNA 6000 Nano Kit) according to the amplitude and sharpness of the peaks corresponding to the rRNA.

RNA sequencing and differential gene expression analyses were performed by SeqCenter (Pittsburgh, Pennsylvania, USA). cDNA libraries were prepared with Stranded Total RNA Prep Ligation with Ribo-Zero Plus kit (Illumina) and 10 bp unique dual indices. Sequencing was performed on a NovaSeq X Plus, yielding pair-ended 150 bp reads. Demultiplexing, quality control and adaptor trimming were performed with bcl-convert (va.2.4, Illumina). Read mapping was performed against the reference genomes (GenBank accession numbers NZ_CP008706.1 and NC_011586.2 for AB5075 and AB0057, respectively) using HISAT2 (v2.2.1, default parameters + ‘--very-sensitive’)^82^, and read quantifications were obtained using Subread (v2.0.6, featureCounts function, default parameters + ‘-Q 20’)^83^. Read counts were loaded into an R environment and edgeR (v1.14.5, Trimmed Mean of M Values, default parameters) was used for normalisation. edgeR was further used for differential gene expression analysis (glmQLFTest, default parameters)^84^. Genes with |logFC | > 1.0 and *p*-value < 0.05 were considered significantly differentially regulated (Supplementary Table S1, Supplementary Table S2). Statistical analysis was performed using edgeR’s exact test for differences between two groups of negative-binomial counts. Volcano plots were generated with VolcaNoseR^85^.

### Biofilm formation assays

Overnight cultures of the *A. baumannii* strains of interest were adjusted to an OD_600_ 0.05 in LB broth (supplemented with IPTG 1 mM when indicated). 200 µl of the diluted cultures were dispensed in 96-well plates and incubated at 37 °C, 180 rpm for 18 h. Following incubation, the biofilms were stained and quantified as described by de Dios et al.^74^ and Harkova et al.^17^.

### Twitching motility assays

Twitching assays were performed as previously described^17^. Briefly, twitching motility was assayed in twitching agar plates (tryptone 10 g/L, yeast extract 5 g/L, agar 10 g/L, supplemented with IPTG 1 mM when indicated). Twitching plates were inoculated with fresh colonies of the strain of interest grown overnight on LB agar at 37 °C by picking them with a plastic pipette tip and stabbing them in the agar until reaching the Petri dish plastic surface. The plates were incubated at 37 °C for 48 h and the twitching diameter was determined.

### Congo red exopolysaccharide binding assays

A Congo red binding assay was used to measure the effect of high/low c-di-GMP in WT AB5075 and AB0057 and the Δ*pdeD* mutation and complementated mutant in *A. baumannii* AB0057. The strains of interest were grown in LB broth overnight to stationary phase at 37 °C. The cultures were then adjusted to an OD_600_ of 1.0 in PBS. Five-µl aliquots of these cell suspensions were spotted on LB agar plates supplemented with 50 mg/L Congo red and 1 mg/L Brilliant blue^86^. The spots were air-dried in a laminar flow hood and the plates were incubated at 37 °C for 48 h.

### *G. mellonella* in vivo virulence testing

To obtain an initial assessment of the effect of high and low c-di-GMP levels on *A. baumannii* AB5075 and AB0057 virulence, the *G. mellonella* model of infection was used as an in vivo model as described by Harkova et al.^17^ with slight modifications. Overnight cultures of WT AB5075 and AB0057, and the respective derivative strains constitutively expressing either pleD* or *rocR* from a pWH1266-Apr-based plasmid (or an empty vector control) were washed twice in PBS buffer, adjusted to OD_600_ 1.0 and serially diluted to determine the infection inoculum. Ten healthy *G. mellonella* larvae (UK Waxworms Ltd.) of similar size were infected via injection of approximately 10^5^ CFU/larvae of the respective *A. baumannii* strain (or a PBS vehicle control) in the third pair of prolegs. The injected larvae were then incubated at 37 °C for the remaining of the experiment. The survival of the larvae was assessed in 2-h intervals between 22 and 32 h post-infection, with a final assessment at 48 h post-infection. Larvae were deemed dead by a complete lack of response to mechanical stimuli. These experiments were performed in three independent replicates to achieve a total *N* = 30 per strain tested. The probability of survival was assessed using the Long-rank (Mantel-Cox) test on GraphPad Prism.

### DGC and PDE coding gene sequence analysis

To study the sequence conservation of the DGC and PDE coding genes, an in silico analysis was conducted for the presence of functional domains in the respective genes identified by Ahmad et al.^19^ and Guo et al.^20^. Subsequently, all the sequences bearing the domains were obtained from the InterPro database^87^, and an individual Hidden Markow Model (HMM) profile was created for each domain: DGC (IPR050469), PPD esterase (IPR012226) and PPD esterase-like domain (IPR050706). These profiles were then utilised as seeds to search for sequences in a pangenome comprising 8929 *A. baumannii* genomes^88^. The sequences obtained were then classified into the pangenome groups and compared against the reference strains to determine the degree of divergence (percent identity and coverage parameters). To determine the molecular conservation among the strains, we used the molecular phylogeny developed by Moreno-Rodriguez et al.^89^. This phylogeny was constructed using 589 proteins present in 99% of the strains that constitute the pangenome. The phylogeny was represented using the R package ggtree (v1.10.5)^90^.

### PdeD protein structure modelling

The PdeD amino acid sequence (ACJ40048.1) was sourced from the *A. baumannii* AB0057 reference genome (GenBank: NC_011586.2). The PdeD protein structure was predicted using AlphaFold^91,92^ and the resulting structure was visualised on PyMOL (v3.0.3)^93^. Figure 4B was created using BioRender.

### *E. coli* swimming assays

Swimming motility assay was performed as previously described^94^. Briefly, *E. coli* strains were cultured and OD_600_-adjusted as described above for Congo red staining assays. The suspensions were stabbed into the middle of swimming agar plates (1% (w/v) tryptone, 0.5% (w/v) NaCl, 0.25% (w/v) agar) using a sterile pipette tip. The swimming media were supplemented with 0.15% arabinose and 100 mg/L ampicillin. Plates were incubated at 37 °C for 16 h and the swimming diameter was measured.

### Growth curves

Growth of WT AB0057, the Δ*pdeD* mutant and the complemented mutant was monitored in LB or M9 minimal medium supplemented with 0.2% casamino acids and either 0.4% glucose or 0.4% glycerol as described previously^44^. Strains were cultured overnight in LB, washed twice in PBS, and the OD_600_ was adjusted to 0.01 in the corresponding medium. Growth was measured by OD_600_ determination every 30 min for 20 h with a BioScreen C Analyzer at 37 °C with continuous shaking. The area under the curve of each growth curve was used to quantify growth and to compare it between strains^95^.

### Mucoviscosity measurements

Mucoviscosity assays were performed as described by Bachman et al.^48^, with slight modifications. Bacteria were grown on LB agar for 24 h at 37 °C. The biomass was collected and washed twice with PBS, and the OD_600_ was adjusted to 4.0 in a total volume of 10 mL. The bacterial suspensions were then centrifuged at 1000 × *g* for 5 min and the OD_600_ of the supernatant, taken from the middle of the tube, was measured. Mucoviscosity was measured as the percentage of sedimentation by the formula S (OD_600_%) = OD_600_(post-spin) / OD_600_(pre-spin).

### Colloidal gradient-based capsule production assessment

To perform a qualitative assessment of the capsule production of the Δ*pdeD* mutant compared to the WT AB0057 and the complemented mutant, we performed a density gradient-based capsule test as described by Valcek et al.^49^, with slight modifications. Briefly, the strains of interest were grown overnight in LB broth at 37 °C and 5 OD_600_ units of biomass were centrifuged at 7000 × *g* for 2 min. The supernatant was completely removed, and the biomass pellets were resuspended in 1 mL PBS buffer. 875 µL of each of these cell suspensions were mixed with 125 µL of LUDOX LS colloidal silica 30% (Merck). The mixtures were then centrifuged at 12,000 × *g* for 30 min at room temperature, and the tested samples were immediately photographed and compared. A representative image of each comparison from three biological replicates is shown.

### Capsule polysaccharide profiling

Capsular polysaccharides were extracted as previously described^96,97^. Briefly, the bacterial strains were grown on LB agar for 24 h at 37 °C. The bacterial biomass of each tested strain was scraped off the plate and resuspended to an OD_600_ of 0.6. 1 ml of the suspension was pelleted, then resuspended in lysis buffer (60 mM Tris-HCl pH 8, 10 mM MgCl_2_, 50 µM CaCl_2_, 3 mg/ml lysozyme, 60 U/ml DNase, and 10 µg/ml RNase) and incubated at 37 °C for 1 h, followed by three freeze/thaw cycles. An additional DNAse and RNAse treatment was performed for 30 min at 37°C. Subsequently, the samples were treated with 10% SDS at 37 °C for 30 min, then incubated with proteinase K (40 µg) at 60 °C for 1 h. The samples were centrifuged at full speed for 2 min at room temperature. The supernatants were then collected, and polysaccharides were precipitated overnight at –20 °C with 400 µL of cold 75% ethanol. The polysaccharides were collected by centrifugation and air-dried. The dried pellets were resuspended in SDS sample buffer, boiled, and loaded and separated on a SurePAGE, Bis-Tris, 4–12% gel (GenScript). Gels were stained for 60 min with 0.1% (w/v) Alcian blue and imaged with a ChemiDoc™ Touch Imaging system (Bio-Rad).

### UPLC/MS-based c-di-GMP quantification

C-di-GMP quantification by UPLC/MS was conducted as previously described^98^. Briefly, overnight cultures of the strains of interest were diluted 1/100 in fresh LB broth, and grown to late-log phase (OD_600_ = 1.0) at 37 °C. For the inducible *E. coli* strains, LB was supplemented with 0.15% L-arabinose. Following incubation, 5 mL were collected by centrifugation, and the intracellular nucleotides were extracted by resuspending the pellet with 100 µl of extracting buffer (40:40:20 (methanol:acetonitrile:0.1 N formic acid in water)). The slurry was incubated for 30 min at –20 °C, and the soluble fraction was collected after a 5 min centrifugation at 16,000 × *g* at 4 °C. This fraction was collected and dried using a SpeedVac vacuum concentrator. The samples were then resuspended in 100 μL of HPCL-grade water, and 10 μL of each sample was analysed on an Acquity Ultra Performance LC system coupled with a Quattro Premier XE mass spectrometer. Chromatography and multiple reaction monitoring parameters were performed as previously described^98^. A c-di-GMP standard curve was determined with the following concentrations: 1.9, 3.9, 7.8, 15.6, 31.3, 62.5, and 125 nM. Intracellular concentrations of c-di-GMP for each sample were calculated by dividing the total moles of c-di-GMP by the product of the respective CFU counts and considering a standard cell volume of 6.36 × 10^–16^ L^98^.

### ELISA-based c-di-GMP quantification

To obtain samples for c-di-GMP quantification by enzyme-linked immunosorbent assay (ELISA), the strains of interest were cultured as described above for biofilm formation, and the bacterial pellets were lysed in Bacterial Protein Extraction Reagent (B-PER; ThermoFisher Scientific). The resulting soluble fractions were used for c-di-GMP quantification using the c-di-GMP ELISA kit (Cayman Chemical, Michigan, USA) according to the manufacturer’s recommendations.

### Resistance to the disinfectants benzethonium chloride (BZT) and chlorhexidine (CHG)

The resistance to BZT and CHG was performed as described by Tipton et al.^64^ with slight modifications. Briefly, the bacterial strains were grown in LB broth overnight at 37 °C at 200 rpm. The cultures were diluted 1:100 in fresh LB medium and grown to mid-log phase (OD_600_ of 0.6). Cells were washed three times in LB broth, and 10^7^ CFU/ml were incubated in the presence of 67 µM BZT and 40 µM CHG at room temperature. At 30 min post-incubation, bacterial survival was determined by serial dilution and enumeration on LB agar.

### Desiccation tolerance assays

Tolerance to desiccation was performed as described by Farrow et al.^53^ with slight modifications. Briefly, overnight cultures were washed twice with bi-distilled water and resuspended to a cell concentration of approximately 10^9^ CFU/ml. 10 µl of each suspension were spotted in the wells of a polystyrene 96-well plate (flat bottom) and were air-dried in a biosafety cabinet. Once visibly dried, the samples were placed in the dark at room temperature and ambient humidity. Cell viability/cultivability was determined at days 7 and 14 after desiccation by rehydrating each sample with 100 µl of PBS for 30 min at room temperature, followed by serial dilution and plating on LB agar. A non-desiccated control was resuspended, serially diluted and plated on LB agar right after the samples were visibly dried.

### Human serum resistance assays

Growth of *A. baumannii* AB0057 and derivative strains in human serum was performed as previously described^44^. Briefly, bacteria were cultured overnight in LB broth at 37 °C. Bacterial cultures were diluted 1:100 in fresh medium and grown to mid-log phase (OD_600_ 0.6) at 37 °C. Bacteria were washed with PBS, and 10^7^ CFU/mL were incubated either with 90% normal human serum or 90% heat-inactivated serum (Innovative Research). Suspensions were incubated at 37 °C, and viable cell counts were determined at 0, 1, 2, and 3 h post-incubation by serial dilution and plating on LB agar.

### Hydrogen peroxide and hypochlorite resistance assays

Resistance to H_2_O_2_ and sodium hypochlorite was performed as previously described^99^, where mid-log phase cultures (OD_600_ 0.6) were spread on LB agar with a swab and allowed to dry for 30 min. Then, a filter paper disk (7 mm diameter; Beckton Dickinson) was added to the middle of the plates and 10 μl of hydrogen peroxide (30%) or sodium hypochlorite (5%) were added to the filters. The plates were incubated overnight at 37 °C, and the diameter of inhibition zones was measured.

### A549 cell adhesion assays

Adhesion to human alveolar basal epithelial cells A549 (ATCC CCL-185) was performed as previously described^44^. Briefly, cells were cultured to confluence in 24-well plates in Kaighn’s Modification of Ham’s F-12 Medium (ATCC 30-2004) supplemented with 10% heat-inactivated foetal bovine serum at 37 °C and 5% CO_2_. Bacterial strains were cultured overnight in LB, washed twice with PBS and adjusted to 10^7^ CFU/ml in Kaighn’s Modification of Ham’s F-12 Medium supplemented with 10% heat-inactivated FBS. Bacterial suspensions were added to each well at an MOI of 10. Bacterium-host cell contact was enhanced by a 5-min centrifugation at 600 × *g*. At 2 h post-incubation, cells were washed 3 times with DPBS (removing the non-adherent bacteria), lysed with 0.25% Triton X-100 for 5 min, and serially diluted for CFU enumeration. Quantification of cell-associated bacteria was performed as previously described^100^.

### Intramacrophage survival

Intramacrophage survival was performed as described by Subashchandrabose et al.^101^, with slight modifications. Briefly, mouse macrophage cell line RAW 264.7 was grown in DMEM supplemented with 10% FBS, 5% L-Glutamine, 1% NEAA, and 1% HEPES^102^. Macrophages were infected with bacteria at an MOI of 100 from bacteria cultured overnight. Bacteria-macrophage interaction was enhanced by a 5-min centrifugation at 500 × *g*. To allow phagocytosis, the plates were incubated for 30 min at 37 °C with 5% CO_2_, then the cells were washed three times with DPBS, and amikacin (150 μg/ml) was added to kill extracellular bacteria. Intramacrophage survival was determined at 30 min post-amikacin treatment by lysing the macrophages with 0.25% Triton X-100, and intracellular bacteria were enumerated on LB agar.

### Murine model of bacteraemia

All infections performed in this study were mono-infection. Mice infections were performed as described previously^44,55^, in which female CBA/J mice (*N* = 10) aged from 6- to 8-weeks-old were inoculated via tail vein injection with 10^7^ CFU. Neutrophils were depleted by intraperitoneal injection of 100 µg of rat anti-mouse monoclonal antibody (MAb) RB6-8C5 (RB6) (BioXCell) 24 h prior infection^44,103,104^. At 24 h postinfection, mice were euthanised, and the spleen, liver and kidneys were aseptically removed and homogenised. Homogenates were serially diluted and plated on LB agar to determine the bacterial burden in these organs.

### Ethics statement

All procedures involving the use of mice were approved by the University Committee on Use and Care of Animals (UCUCA) of the University of Michigan Medical School (protocol # PRO00007111), in accordance with the Institutional Animal Care and Use Committee (IACUC), the Office of Laboratory Animal Welfare (OLAW) and the United States Department of Agriculture (USDA). The protocol was accredited by the Association for Assessment and Accreditation of Laboratory Animal Care, International (AAALAC, Intl.), in accordance with the Guide for the Care and Use of Laboratory Animals (8th edition) of the National Research Council of the National Academies. Mice were euthanized by inhalant anaesthetic (isoflurane) overdose followed by vital organ removal.

### Statistical analysis

Graphs show average values ± SD (standard deviation). Data representation and statistical analyses were performed on GraphPad Prism 10.4.1 (627) (GraphPad Software, San Diego, California USA, www.graphpad.com). For each experiment, the corresponding number of independent replicates and statistical analysis, including post hoc corrections, are indicated in the respective figure legends. Parametric or non-parametric tests were selected depending on the results of normality tests performed on each individual dataset.

## Supplementary information

Supplementary materials

Supplementary Table S1

Supplementary Table S2

Supplementary Table S3

Supplementary Table S4

## Data Availability

RNA-seq datasets generated in this work have been deposited in the Gene Expression Omnibus repository under the accession numbers GSE229819 and GSE291750 for *A. baumannii* AB5075 and *A. baumannii* AB0057, respectively.

## Code availability

No code was generated for this study.
