# Supplementary material for: Network-level divergence in cyclic di-GMP signalling drives ecological versatility in Acinetobacter baumannii
Source: NPJ Biofilms Microbiomes. 2026 Apr 9;12:112. doi: 10.1038/s41522-026-00968-y (PMC13243610; doi:10.1038/s41522-026-00968-y)
Supplement: Supplementary file 1 — Supplementary materials [file 41522_2026_968_MOESM1_ESM.pdf]

Network-level divergence in cyclic di-GMP signalling drives ecological versatility in *Acinetobacter baumannii*

Rubén de Dios, Valerie Forsyth, Lyuboslava G Harkova, Kylie Schache, Brian Hsueh, Hannah Strat, Brynn Riley, Alejandro Rubio, Antonio J Pérez-Pulido, Christopher Waters, Harry LT Mobley[2], Sébastien Crépin and Ronan R McCarthy

Supplementary Information

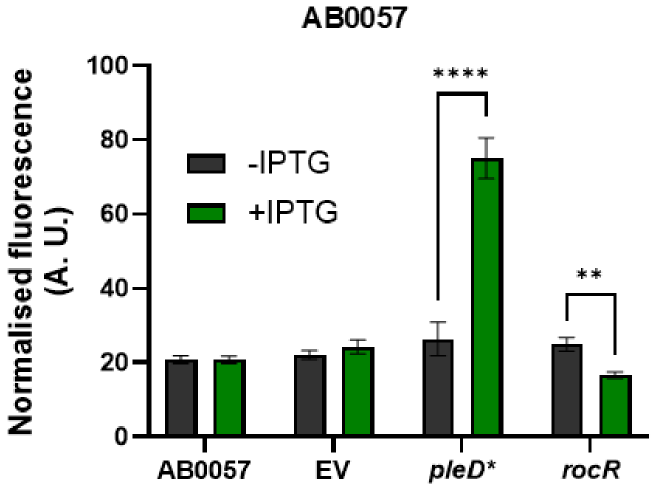

Supplementary Figure S1. Similarly to our previous report using AB5075 (Harkova *et al*., 2024), the intracellular levels of c-di-GMP in AB0057 could be controlled via IPTG-induced expression of the DGC *pleD** (increased c-di-GMP levels) or the PDE *rocR* (decreased c-di-GMP levels). C-di-GMP levels in this strain, compared to WT and empty vector controls (EV), were quantified using the fluorescent CensYBL-Ab c-di-GMP biosensor (Harkova *et al*., 2024). Averages ±S.D. from five biological replicates are represented. Statistical analysis was done by two-way ANOVA (Sidak's correction). Statistical significance is indicated as **p<0.01; ****p<0.0001.

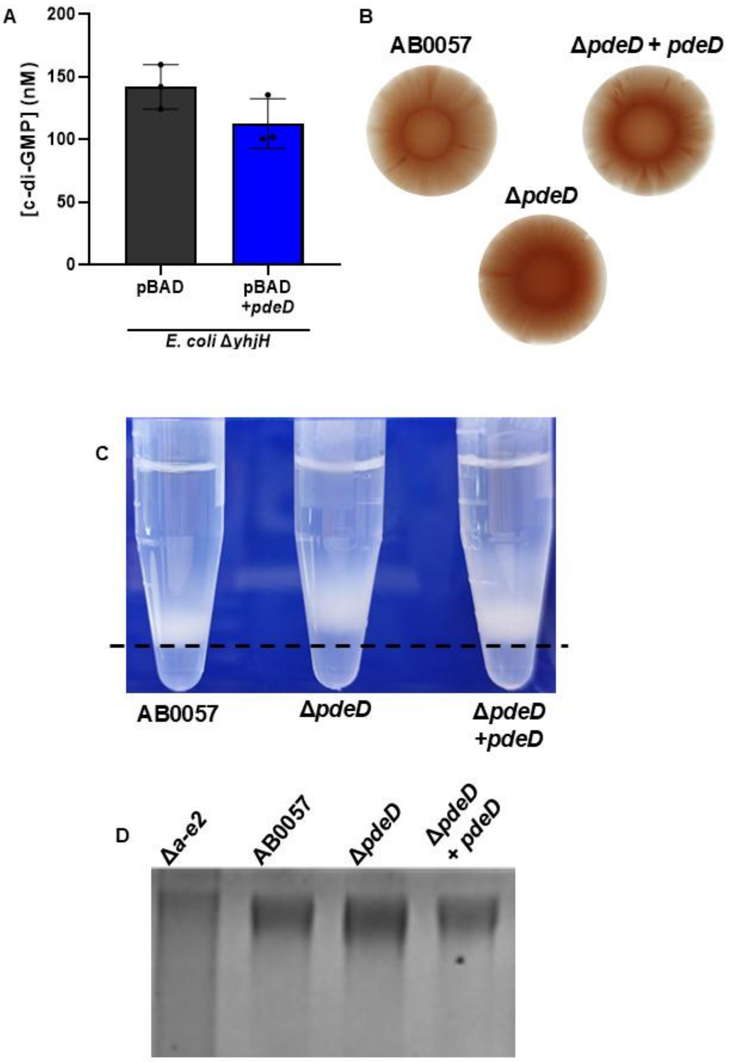

Supplementary Figure S2. The expression of *pdeD* in an *E. coli* Δ*yhjH* mutant reduces the levels of c-di-GMP compared to the empty vector control (pBAD) and increases EPS and capsular polysaccharide production. A) After the identification of PdeD through the swimming-based functional screening (Figure 4A) as the only *A. baumannii* AB0057 enzyme able to complement this phenotype in a Δ*yhjH* mutant, we quantified the levels of c-di-GMP in the *pdeD*-expressing strain with respect to the empty vector control through UPLC/MS. Averages ±S.D. from three biological replicates are represented. **B**) A Congo red assay showed an increased production of exopolysaccharide in a colony biofilm in the absence of *pdeD* compared to a wild type AB0057 and the complemented mutant**.** The photographs shown are representative images of three biological replicates. **C**) Capsule production was qualitatively assessed by a density gradient-based method (Philippe *et al*., 2022). The Δ*pdeD* mutant showed a lower density (did not sediment as much as the WT and complemented strains), which is indicative of a greater capsule production. The dashed line indicates the sedimentation level of the WT AB0057. One representative photograph from three biological replicates is shown. **D**) The increased capsule production in the Δ*pdeD* mutant compared to the wild type AB0057 and the complemented mutant was also directly visualised by capsular polysaccharide purification and SDS-PAGE after staining with alcian blue**.** The non-capsulated Δ*gna-gne2* mutant (Δ*a-e2*) was used as negative control. The gel presented is a representative image out of three biological replicates.

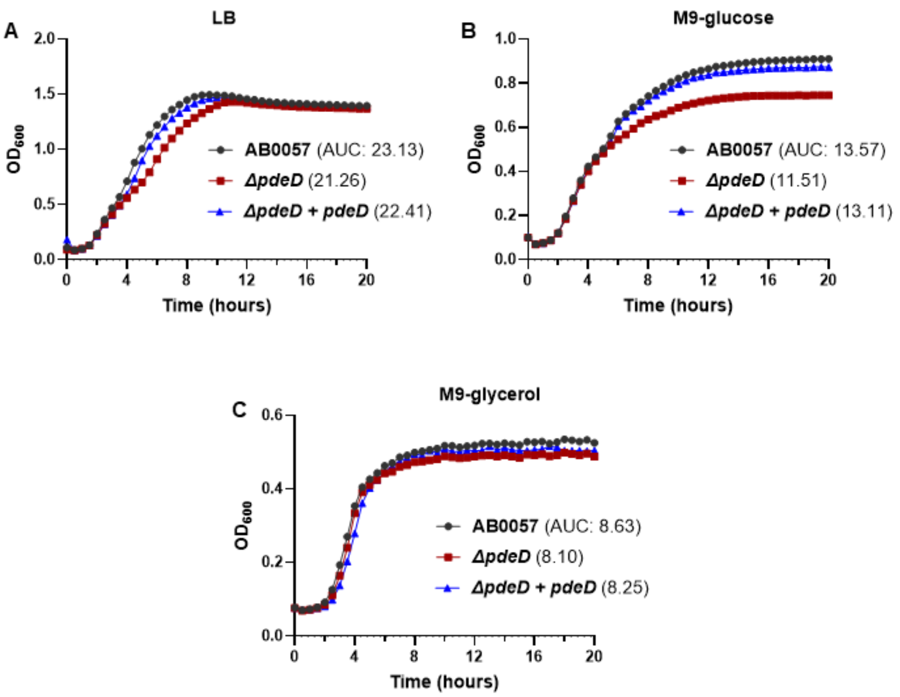

Supplementary Figure S3. Growth of the Δ*pdeD* mutant compared to the WT *A. baumannii* AB0057 and the complemented mutant. The growth was measured in rich LB medium (**A**) and in M9 minimal medium with 0.4% glucose (**B**) or 0.4% glycerol (**C**) as carbon source. For each growth curve, the area under the curve (AUC) was calculated to quantify growth and compare between strains. OD_{600} was measured every 30 minutes for 20 hours at 37 °C with continuous shaking. Each data point represents the average ±S.D. from four biological replicates.

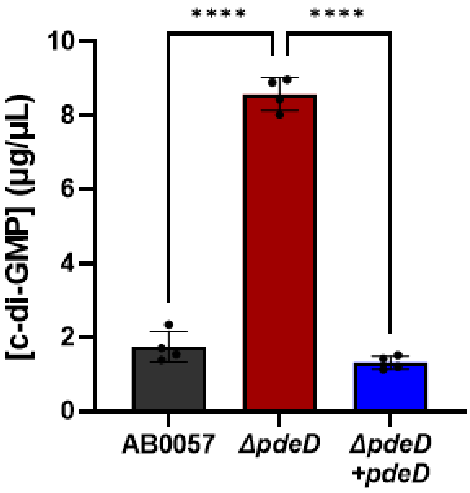

Supplementary Figure S4. The Δ*pdeD* mutant shows higher c-di-GMP levels that the WT *A. baumannii* AB0057 and the complemented mutant strain. To confirm the UPLC/MS c-di-GMP quantifications performed in these strains, we also quantified the levels of this second messenger using an enzyme-linked immunosorbent assay (ELISA)-based c-di-GMP quantification kit. Averages ±S.D. from four biological replicates are represented. Statistical analysis was performed by one-way ANOVA (Tukey's correction). Statistical significance is indicated as ****p<0.0001.

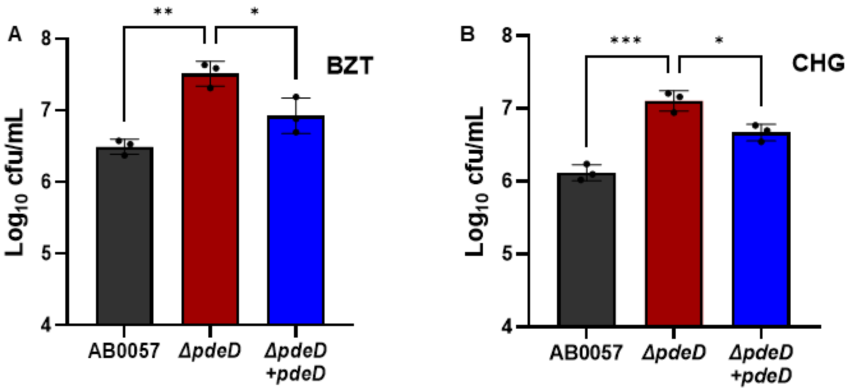

Supplementary Figure S5. The Δ*pdeD* mutation alters the resistance of *A. baumannii* AB0057 to disinfectants that disrupt the bacterial cell membrane. The tolerance of the Δ*pdeD* mutant to two common disinfectants such as benzethonium chloride (BZT) (**A**) and chlorhexidine gluconate (CHG) (**B**) was also assessed, indicating an increased tolerance compared to the WT AB0057 and the complemented mutant. Averages ±S.D. from three biological replicates are represented. Statistical analysis was done by one-way ANVA (Tukey's correction).
